# Characteristics and Impact of the rNST GABA Network on Neural and Behavioral Taste Responses

**DOI:** 10.1523/ENEURO.0262-22.2022

**Published:** 2022-10-04

**Authors:** Susan P. Travers, B. Kalyanasundar, Joseph Breza, Grace Houser, Charlotte Klimovich, Joseph Travers

**Affiliations:** Division of Biosciences, College of Dentistry, Ohio State University, Columbus, OH 43210

**Keywords:** DREADDs, inhibitory interneurons, optogenetics, solitary nucleus, taste quality coding

## Abstract

The rostral nucleus of the solitary tract (rNST), the initial CNS site for processing gustatory information, is comprised of two major cell types, glutamatergic excitatory and GABAergic inhibitory neurons. Although many investigators have described taste responses of rNST neurons, the phenotypes of these cells were unknown. To directly compare the response characteristics of both inhibitory and noninhibitory neurons, we recorded from mice expressing Channelrhodopsin-2 (ChR2) under the control of GAD65, a synthetic enzyme for GABA. We observed that chemosensitive profiles of GABAergic taste neurons (G+_TASTE_) were similar to non-GABA taste neurons (G-_TASTE_) but had much lower response rates. We further observed a novel subpopulation of GABA cells located more ventrally in the nucleus that were unresponsive to taste stimulation (G+_UNR_), suggesting pathways for inhibition initiated by centrifugal sources. This preparation also allowed us to determine how optogenetic activation of the rNST GABA network impacted the taste responses of G-_TASTE_ neurons. Activating rNST inhibitory circuitry suppressed gustatory responses of G-_TASTE_ neurons across all qualities and chemosensitive types of neurons. Although the tuning curves of identified G-_TASTE_ were modestly sharpened, the overall shape of response profiles and the ensemble pattern remained highly stable. These neurophysiological effects were consistent with the behavioral consequences of activating GAD65-expressing inhibitory neurons using DREADDs. In a brief-access licking task, concentration-response curves to both palatable (sucrose, maltrin) and unpalatable (quinine) stimuli were shifted to the right when GABA neurons were activated. Thus, the rNST GABAergic network is poised to modulate taste intensity across the qualitative and hedonic spectrum.

## Significance Statement

We used GAD65-Channelrhodopsin-2 (ChR2) mice to demonstrate, for the first time, that rostral nucleus of the solitary tract (rNST) GABAergic (GAD65) neurons respond to taste stimuli. Chemosensitive profiles of GABA taste neurons were similar to those of non-GABA cells, but their overall responses were weaker. Other GABA neurons were unresponsive to oral stimuli, implying they are targets for centrifugally-initiated inhibitory influences. In non-GABA neurons, activating the rNST GAD65 network profoundly suppressed the gain of taste responses elicited by all qualities, but left coding largely unaltered. In behaving animals, activating GAD65 neurons with DREADDs produced rightward shifts in preference for sucrose and avoidance of quinine. We hypothesize that rNST GABA neurons are important in changing the salience of gustatory signals in response to varying homeostatic or cognitive demands.

## Introduction

The rostral nucleus of the solitary tract (rNST), the initial CNS site for processing gustatory information, contains two major neuronal populations: excitatory, glutamatergic cells that often project outside the NST, including the parabrachial nucleus (PBN; Gill et al., 1999), and GABAergic inhibitory neurons, many consisting of local interneurons (Lasiter and Kachele, 1988; Davis, 1993). The function(s) of these inhibitory neurons are still obscure, but one early report provided evidence that they are a necessary link for suppressive influences on rNST taste responses that originate in the cortex (Smith and Li, 2000). The sensory properties of solitary nucleus GABA cells are largely unknown. Although there are numerous *in vivo* recordings from single rNST taste neurons, the neurotransmitter phenotypes corresponding to these responses were not identified. Thus, gustatory responses of glutamatergic and GABA neurons have never been directly compared, although hints can be gleaned from contrasting rNST cells projecting to PBN (presumed glutamatergic) versus those that do not (presumed GABAergic). Based on antidromic activation, PBN projection cells typically exhibit more vigorous taste responses, although both categories respond to a range of taste qualities (Ogawa et al., 1984; Monroe and Di Lorenzo, 1995; Cho et al., 2002; Geran and Travers, 2009). Nevertheless, whether the nonantidromically activated neurons were GABAergic is ambiguous. Instead, these cells may have been a different interneuron type or have projected to other rNST targets such as the reticular formation or caudal, visceral NST (JB Travers and Travers, 2018). Indeed, even whether GABA neurons respond to taste stimulation has been uncertain. In the present report, we used transgenic mice expressing Channelrhodopsin-2 (ChR2) under the control of GAD65, a synthetic enzyme for GABA. This allowed us to use “optotagging” (Lima et al., 2009; Deubner et al., 2019) in an *in vivo* preparation to identify GABAergic taste cells and directly compare their characteristics to non-GABA taste cells. The expression of ChR2 in GABAergic neurons further allowed evaluation of how responses of non-GABA neurons are influenced by activating the rNST GABA network which is comprised of synapses from a large population of local inhibitory interneurons as well as GABA terminals from the cNST (S Travers et al., 2018) and the central nucleus of the amygdala (Saha et al., 2000, 2002; Bartonjo and Lundy, 2020; Jin et al., 2021).

In other CNS regions, different populations of GABA interneurons have diverse effects on sensory responses, broadly categorized as “divisive,” mainly impacting response gain, or “subtractive,” sharpening tuning (Wilson et al., 2012). In the NST, a classic *in vivo* experiment reported that local infusion of GABA_A_ receptor antagonists gave rise to broader tuning profiles (Smith and Li, 1998), suggesting subtractive influences. However, in that study, response profiles were only partially evaluated and the phenotype of the neurons that were recorded were unknown. In contrast, using the mouse model employed in the present study, an *in vitro* experiment evaluated effects of optogenetic release of GABA on afferent-evoked responses in non-GABA neurons and posited a mostly divisive inhibitory effect based on analyzing response curves elicited by solitary tract stimulation at different frequencies (Chen et al., 2016). In the current experiment, we came to the same conclusion by (1) evaluating effects of inhibition on taste response profiles of non-GABA neurons using natural taste stimulation, and (2) in a subset of neurons, eliciting responses of different magnitude using oral optogenetic stimulation at different frequencies (Baumer-Harrison et al., 2020) parallel to the *in vitro* study. In both cases, the main effect of inhibition was on response gain. Although tuning curves became modestly sharper using natural stimulation, there was a marked suppression in response magnitude that extended to all qualities and neuron types. Importantly, the ensemble code for taste quality remained highly stable. These neurophysiological effects were echoed in a separate behavioral study. Using a brief-access licking task, activating local GAD65-expressing neurons with DREADDs shifted response-concentration curves for both palatable and unpalatable stimuli to the right but preserved the appropriate behaviors of acceptance and rejection.

## Materials and Methods

### Neurophysiology

#### Mice

**Figure 1.**
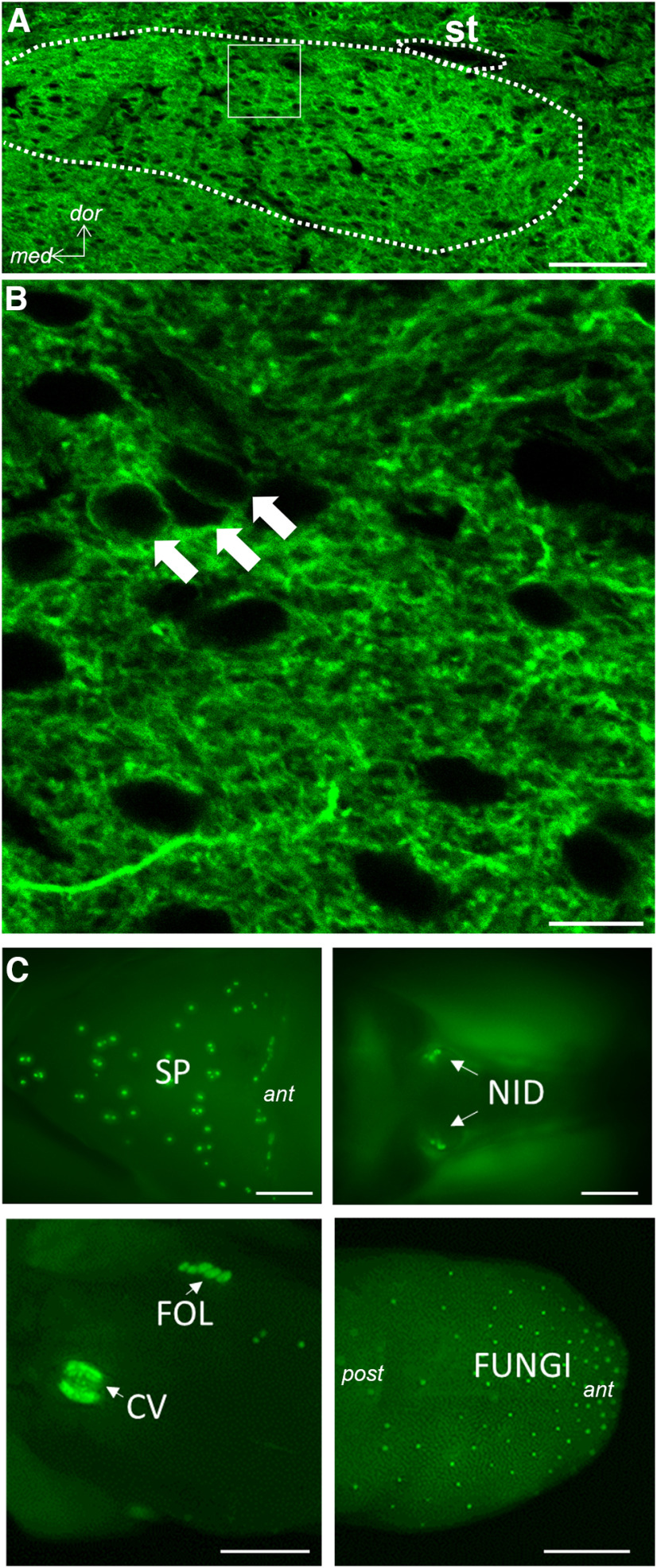
Expression of EYFP in the rNST and taste buds of a GAD65-ChR2/EYFP mouse. ***A***, 20× confocal photomicrograph (single 2-μm Z level) demonstrates EYFP expression throughout the nucleus. ***B***, At higher magnification (60×, digital zoom = 3, single 1-μm z level), EYFP expression is evident in somal membranes (arrows) and the neuropil. Scale bars: 100 μm (***A***) and 10 μm (***B***). White dotted line indicates the approximate border of the nucleus. st, solitary tract; dor, dorsal; med, medial. ***C***, Low-power photomicrographs of native EYFP expression in different taste bud groups: soft palate (SP), nasoincisor ducts (NID), circumvallate (CV), foliate (FOL), and fungiform papillae (FUNGI). These are surface views of the whole tongue from above and the peeled palatal epithelium, viewed from below. In each panel, anterior (ant) is to the right and posterior (post) to the left. Scale bars: 1 mm. Extended Data Figure 1-1 provides functional characterization of these taste bud cells. Immunohistochemistry shows that EYFP/ChR2 taste bud cells in this mouse are mostly Type I cells. Activating them with blue light elicits NST responses dependent on oral P2X3-containing receptors, similar to responses to taste stimuli.

All procedures involving animals were approved by the Ohio State University IACUC. Most mice expressed ChR2 and EYFP under the control of the GAD65 promoter (*N* = 66). These “GAD65-ChR2/EYFP” mice were generated by crossing a GAD65-cre line (Jax 010802) with mice carrying a floxed ChR2/EYFP allele (JAX 012569 or 024109). This cross yielded expression of the fluorescent protein throughout the brain, including the entire NST. Expression was copious in the neuropil, but also evident in soma when inspected with confocal microscopy (Fig. 1*A*,*B*). Three mice were generated from a cross of the same ChR2 mice with a strain expressing cre under the control of VGAT (Jax 016962). Thus, all mice expressed ChR2 in GABAergic inhibitory neurons. The strains were pooled for analysis since there were no obvious differences in response properties or effects between them. Both males and females were used (27 females, 39 males, 3 unknown) and the results from both sexes were combined in the analyses presented below since inspection of the data yielded no notable differences in the maximum response rates, the degree of inhibition or the proportion of taste-responsive neurons directly driven by brain light between the two sexes (all *p*s > 0.1 based on *t* tests and χ^2^ evaluation). Mice were adults and ranged in age from 42 to 302 d (mean = 137.4 ± 7.2 d).

10.1523/ENEURO.0262-22.2022.f1-1 Extended Data Figure 1-1 Most taste bud cells in the GAD65-cre X ChR2/EYFP cross are Type I cells. Activating taste bud cells in GAD65-ChR2/EYFP mice evokes responses in NST taste neurons that are dampened by lingual application of a P2X3 antagonist. ***A***. Immunostaining of taste buds from GAD65-ChR2/EYFP mice. Mice were perfused with phosphate-buffered saline followed by paraformaldehyde/lysine-metaperiodate and 40 μm frozen sections of fungiform and circumvallate papillae cut on a sliding microtome. Standard double-labeling immunofluorescent techniques with appropriate antibodies were used to identify Type I (NTPDase -Sevigny, host: rabbit, RRID: AB_2314986, 1:1000), Type II (PLCβ2 - Santa Cruz, host: rabbit, RRID: AB_2314986, 1:50 and Type III cells (carbonic anhydrase 4 [CA4] - R&D Systems, host: goat, RRID: AB_2070332, 1:1000). Analysis was performed by inspecting 1 μm confocal z-stacks for each of the three stains (PLCβ2 - 52 cells from 2 fungiform & 8 circumvallate papillae; CA4 - 60 cells from 4 fungiform & 6 circumvallate papillae; NTPDase - 3 fungiform & 7 circumvallate buds - individual cells not counted. None of the PLCβ2- or CA4-labeled cells co-expressed EYFP. In contrast, there was extensive co-labeling of taste bud cells stained for NTPDase suggesting that most ChR2/EYFP-expressing cells in this mouse line are Type I cells (Baumer-Harrison et al., 2020; Larson et al., 2021; Rodriguez et al., 2021). Scale bar= 10 μm. ***B***. Ionotropic P2X3 receptors are involved in conveying mouth-light driven responses centrally to a similar degree as taste responses. Multiunit NST recordings from the GAD65-ChR2/EYFP mouse (same strain as in the main part of the study). The data is from a separate series of experiments using similar techniques to record multiunit responses to probe the role of ionotropic P2X3 receptors in conveying light_m_ responses centrally. Ionotropic P2X2/P2X3 heterodimers (and some homodimers) are present on primary afferent taste neurons and responsible for transmitting responses from taste buds to the primary afferent nerves (Finger et al., 2005). The marked suppression of responses to both light_m_ and taste stimuli after topical application of a P2X3 antagonist suggests a common mechanism in conveying responses to the primary afferents, although this effect could also reflect crucial signaling within the bud (Rodriguez et al., 2021). Bar graphs depict the mean magnitude of multiunit responses to a taste mixture, individual tastants and light directed at the oral cavity (5 ms/10hz/10mW) through a 400 μm optical fiber. To quantify responses, neural activity was integrated (0.1 s time constant) and then the area under the curve during the 10 s stimulation period was summed and adjusted by activity during the immediately preceding baseline. Responses were normalized to the maximum control response in a given mouse. Responses are shown prior to (control) and following delivery of a vehicle (polyethylene glycol- PG, 10%) and a P2X3 antagonist (AF353 [Alomone Labs, AF-353], 1 mm dissolved 10% PG, (Vandenbeuch et al., 2015). Data are from 6 mice (individual mice denoted by dots). Durations of PG and PG + AF353 application were 18±1 and 20±1 minutes. Stimulus abbreviations and concentrations: SUC: sucrose (300 mm), MSGai: (100 mm MSG, 2.5 mm IMP and 100 μm amiloride), NaCl: (100 mm), CIT: citric acid (10 mm), and BIT: a bitter cocktail of cycloheximide, (10 μm) and quinine monohydrochloride (2.7 mm). ANOVA: significant effects for condition (*p* = 5.55e-06), stimulus (*p* = 2.73e-06) and a condition X stimulus interaction (*p* = 8.77e-05). Pairwise ANOVA comparisons between conditions indicated significant differences between the control and post-AF (*p* = .001) and vehicle vs post-AF (*p* = 8.5e-05) but not between the control and vehicle conditions (*p* = .803). ANOVAs were done without responses to the bitter cocktail due to a missing data point for the post-AF condition. Significant differences between post-PG (vehicle) and post-AF conditions based on Bonferroni-adjusted *t*-tests are indicated by asterisks and p values above the bars. Responses to the bitter stimulus were analyzed separately based on 5 instead of 6 cases. Download Figure 1-1, TIF file.

#### Surgical preparation for recording

To prepare mice for recording, animals were injected with urethane (1 g/kg, i.p.). Throughout surgery, the level of anesthesia provided by this single dose of urethane was supplemented with isoflurane (<1% in O_2_) or sodium pentobarbital (∼25 mg/kg) titrated to achieve an areflexive state. To view the mouth clearly and achieve optimal conditions for fluid and light stimulation, sutures were passed through the maxillary and mandibular lips for retraction, the hypoglossal nerves severed, and a tracheal cannula inserted (Breza and Travers, 2016; Kalyanasundar et al., 2020). We placed the animal in a stereotaxic device, made an incision over the skull and removed a portion of the interparietal plate with a drill and rongeurs to gain access to the rNST. A bolt was glued to the skull between bregma and lambda to stabilize the head without using the mouthpiece, further maximizing our view of the mouth. Following surgery and throughout recording, in most cases (*N* = 63/66), mice were maintained in an areflexive state with isoflurane. In the remaining three subjects, anesthesia was supplemented by sodium pentobarbital or urethane.

#### Taste stimulation

Gustatory stimuli were made with chemicals purchased from Fisher or Sigma and diluted in artificial saliva (AS; in mm: 22 KCl; 15 NaCl, 0.3 CaCl_2_, 0.6 MgCl_2_; Breza et al., 2010). Taste stimuli were delivered from pressurized glass bottles, connected via polyethylene tubes to a manifold (Warner Instruments) attached to a glass tube (1.0–1.2 mm) that was the final common path to deliver fluids to the mouth. In the initial one-third of preparations, the flow rate was ∼0.23 ml/s, but we increased the rate to 0.6 ml/s in later preparations to make it easier to stimulate the whole mouth. Taste stimuli were applied for 10 s, and preceded and followed by the flow of AS. The duration of the AS rinse was at least 20 s and a period of 1 min or more separated successive taste stimulations. Taste stimuli representing the five classic taste qualities were used at mid-high to mid-range concentrations, referred to as stimulus set A and B, respectively (Table 1); the five stimuli were presented in varied order. We began by using the lower concentrations but switched to the higher intensity stimuli because we were interested in evaluating whether inhibition sharpened tuning curves and the initial experiments with the mid-range stimuli revealed many narrowly-tuned units. Thus, we transitioned to the higher concentrations to attempt to broaden response profiles (Wu et al., 2015). In the results below, we use data from neurons tested with either stimulus set for describing general properties of the neurons and characteristics of putative inhibitory NST neurons. However, we restricted analyses to stimulus set A when describing effects of activating NST inhibitory circuitry on putative excitatory taste neurons, which involved more nuanced comparisons of the representation of different qualities under the two conditions. Table 2 details the number of cells tested with each stimulus set.

**Table 1:** Stimuli

Quality	Chemical	Abbreviation	Concentrations (mm)	
			Stimulus set A	Stimulus set B
Sweet	Sucrose	SUC	600	300
Umami	MSG + 2.5 mm IMP + 100 μm amiloride	MSG_ai_	600	600
Salty	NaCl	Na^+^	300	100
Sour	Citric acid	CIT	30	10
Bitter	Cycloheximide + quinine (A) Cycloheximide (B)	BIT	0.01 + 2.7	0.01

MSG, monosodium glutamate; IMP, inosine-5'-monophosphate.

**Table 2:** Stimuli used for taste-responsive cells

Neuron type	Stimulus set	# cells tested	Additional cells with 0.1 m or no umami
G-_TASTE_ (*N* = 84)	A	54*	5
	B	16	9
G+_TASTE_ (*N* = 12)	A	8	2
	B	2	0

*neurons used for analyses of effects of activating the inhibitory network on taste responses.

#### Neural recording and testing protocol

##### Search tracks

During search tracks for locating the gustatory NST, neural activity was recorded through epoxylite-coated tungsten microelectrodes ∼1–3 mΩ (Frederick Haer Inc. or World Precision Instruments). Signals were amplified and filtered (10,000×; 600-10K; Alpha Omega, MCPplus), listened to using an audio monitor (Grass AM8), and displayed on an oscilloscope and computer monitor using AD hardware and software [Cambridge Electronics Design (CED), Spike 2]. We probed for taste-driven NST activity by flowing a mixture of taste stimuli (in mm: 300 sucrose, 10 citric acid, 100 NaCl, and 0.01 cycloheximide) and individual tastants over the entire oral cavity. Initial coordinates were typically 2.5 mm caudal to lambda and 1.0 mm lateral to the midline. Because the GAD65-ChR2 strain expresses ChR2 in taste buds throughout the oral cavity (Fig. 1*C*), prominently in Type I (Extended Data Fig. 1-1*A*; Baumer-Harrison et al., 2020), but perhaps also weakly in other taste bud cell types (Larson et al., 2021) the search for the gustatory NST was facilitated by directing short pulses (5 ms, ∼1–10 Hz) of blue light (473 nm; 10 mW) to oral regions populated by taste buds using an LED source (Thor Labs, DC4104) controlled by CED hardware, CED software, a Grass S48 stimulator, and delivered though a 400 μm, 0.39 NA optical fiber (hereafter referred to as “light_m_,” i.e., light to the mouth). The light_m_ stimulus elicited robust responses in virtually all taste neurons regardless of the quality they were responsive to. In a separate series of multiunit experiments using the GAD65-ChR2 mice we found that these responses were greatly attenuated by topical application of a P2X3 antagonist, AF343, suggesting that taste bud responses from the light_m_ stimulus activate primary afferent fibers requiring the same final common mechanism as natural tastants (Extended Data Fig. 1-1*B*). We also used blunt glass probes or brushes to stroke the oral tissues using non-nociceptive levels of force. Taste-responsive NST locations responded to light_m_ and sometimes to the somatosensory stimulus but solitary nucleus sites purely responsive to the somatosensory stimulus were not activated by mouth light and were typically located lateral to the gustatory-responsive zone.

##### Single-unit recording

After locating the gustatory NST, we switched to recording using an “optrode,” a tungsten electrode (∼2–5 mΩ) combined with a 100 μm, 0.22 NA optical fiber (Thor Labs UM22-100). The optical fiber was configured to terminate <1 mm dorsal to the electrode tip (mean distance = 660 ± 5.7 μm; range 550–820 μm, *N* = 110 measurements). To search for single units, we used taste stimuli and light_m_ and directed blue light pulses (5 ms, 1–10 Hz) to the NST through the optical fiber (hereafter also referred to as “light_br_,” i.e., light to the brain). Light pulses directed through the optrode were generated by a laser (Laserglow, LRS-0473-GFM-00050-05 or LRD-0470-PFFD-00100-05) and controlled by CED software and hardware. Because the laser does not deliver square pulses, measuring a constant light delivered through the optrode does not give an accurate measure of light intensity. Thus, we derived a better estimate using a power meter (Thor PM 100D) to measure the mean light intensity of each pulse within a train of pulses and then averaged across the train. Across the 35 neurons for which this measure was made, pulses were 6.2 ± 0.2mW; the remaining neurons were stimulated using optrodes with similar configurations and laser settings. This brain light stimulus is expected to activate local GABAergic neurons, as well as fibers from GABA neurons outside the NST, notably the central nucleus of the amygdala (Saha et al., 2000, 2002; Bartonjo and Lundy, 2020; Jin et al., 2021) and the caudal NST (S Travers et al., 2018). It is important to note that although GAD65 is expressed in just a subset of GABA neurons in some CNS locations such as the olfactory bulb (Parrish-Aungst et al., 2007), in the rNST it is highly co-localized with VGAT (S Travers et al., 2018), suggesting that it is present in a majority of GABA (and glycinergic) neurons in this location.

When a neuron was located that responded to taste (or mouth light), we screened it to determine whether it also responded to brain light. Cells responding to light_br_ with short latency and in a time-locked, excitatory fashion presumably expressed ChR2 and were likely to be GABAergic. Therefore, we classified them as “G+_TASTE_” neurons, i.e., putative GABAergic taste-responsive neurons. Brain light stimulation consisted of a 10-Hz/10-s train of blue light pulses and/or a series of 20 pulses at 1, 4, 10, 20, and 50 Hz. G+_TASTE_ neurons were then tested with the standard protocol using the classic taste qualities (stimulus set A or B), presented in a varied order, to define their gustatory response profiles. Taste-responsive cells that did not respond in an excitatory, time-locked fashion to light were unlikely to be GABAergic and thus classified as non-GABA taste-responsive neurons (“G-_TASTE_”). This population is liable to include, though not be solely comprised of, glutamatergic neurons, including those that project to the PBN (Gill et al., 1999). G-_TASTE_ cells were tested with the panel of taste stimuli with and without concurrent stimulation with light_br_ (10 Hz) to determine effects of activating the NST GABAergic inhibitory network. Stimulations were repeated whenever possible. If the cell remained isolated, we used a micromanipulator to position the mouth light fiber so that it elicited optimal responses from the cell, and then tested light_m_ responses using nominal frequencies of 2, 5, 10, 20, and 50 Hz for 2 s (because frequency was manually controlled with the Grass Stimulator, actual frequencies deviated slightly: mean ± SEM = 2.3 ± 0.1, 5.3 ± 0.1, 10.5 ± 0.2, 20.5 ± 0.2, 50.1 ± 0.1). Previous work in this same mouse cross (Baumer-Harrison et al., 2020) showed that stimulation of the anterior tongue using light at increasing frequencies produces larger chorda tympani responses that ultimately saturate, similar to effects of increasing tastant concentration (Harada et al., 1997; Frank et al., 2005; Sanematsu et al., 2005). In single gustatory neurons, higher concentrations of sapid stimuli increase firing rate in neurons most sensitive to a given stimulus and can recruit additional responses to less effective stimuli, i.e., make the cells more broadly tuned (Pfaffmann, 1941; Wu et al., 2015). As was done for natural taste stimuli, responses elicited by light_m_ were evaluated in the presence and absence of light_br_. Because a series of light_m_ frequencies could be rapidly tested, we were able to efficiently probe the effects of optogenetic inhibition over a wider range of firing rates.

If a neuron was identified by being driven by light_br_, we first evaluated the characteristics of the response to brain light using the protocol above, then determined whether it was taste (and/or light_m_) responsive. If the cell responded to gustatory stimulation, the chemosensitive profile was determined. If a light_br_-responsive neuron was not activated by taste stimulation and remained isolated, we tested it with oral somatosensory stimuli including gentle stroking of the oral tissues with a blunt glass probe and depressing the mandible. Brain-light responsive neurons unresponsive to taste were classified as “G+_UNR_” neurons (inhibitory unresponsive neurons), unless an oral somatosensory response could be identified, in which case they were deemed “G+_MECH_” (inhibitory mechanically-responsive) cells.

#### Neurophysiological measures

##### Taste and mouth light responses

Responses to both taste and light_m_ stimulation were calculated as evoked activity adjusted by a comparable period without stimulation. Net taste responses were the number of spikes during the 10-s stimulus delivery minus those during the 10-s prestimulus AS period, averaged across trials. The prestimulus period was calculated separately for control stimulations and those with concurrent brain light, since activating the NST inhibitory network often decreased the spontaneous rate of a cell. When stimuli were repeated, the mean of the trials was used. The mean and standard deviation (SD) of the spontaneous and prerinse AS activity were also calculated across trials for each neuron. The response criterion was a net evoked firing rate ≥1 Hz (i.e., at least 10 spikes for 10 s) and 2.5 × the SD of the mean response to AS (Nishijo and Norgren, 1991; Geran and Travers, 2006; Kalyanasundar et al., 2020). However, two neurons that did not strictly meet this criterion were included. One was a bitter-responsive neuron with a clear but delayed response and another responded with a marginal increase in activity (0.9 Hz) with our standard whole-mouth stimulation protocol but responded robustly if stimuli were preferentially directed at the posterior mouth. Net responses to oral stimulation with light were calculated in a similar fashion by summing across the 2-s period of stimulation and adjusting for a comparable unstimulated period. However, in the case of light_m_ stimulation, the net response was adjusted by the spontaneous rate, since AS did not flow during the mouth light stimulation. The criteria for a response to mouth light stimulation was the same as that for a taste response, 2.5 × SD of the spontaneous rate.

##### Brain light responses

To analyze responses to brain light, we triggered off the beginning of the stimulus pulse and set a 10-ms search window for detecting a spike. Latency, jitter (SD of latency) and the proportion of trials evoking a spike were calculated for a given frequency and averaged across any repeated trials.

#### Statistical analyses

Statistical analyses were conducted and graphs prepared in Systat (v13), Excel (2016), and GraphPad Prism (v9.1.1). Comparisons between spontaneous rate, anatomic location, and taste responses for G+_TASTE_, G+_UNR_, G-_TASTE_ neurons were performed using nonparametric or parametric ANOVA. *Post hoc t* tests to assess differences in responses for individual stimuli were Bonferroni-adjusted and Welsh’s correction for unequal sample sizes applied as warranted. Comparisons between net taste responses under control conditions and during brain light stimulation (light_br_) were restricted to neurons tested with stimulus set A and compared using repeated measures or mixed ANOVAs. To identify similarities between response profiles, we used hierarchical cluster analysis based on responses to representatives of the five standard taste qualities, Pearson’s correlations, and an average amalgamation schedule. The scree plot was consulted to determine the number of neuron groups. To evaluate across-neuron patterns of activity, Pearson’s correlations were calculated between stimuli across taste-responsive cells during the control period and the period with simultaneous brain light, then plotted in a multidimensional scaling space. Breadth of tuning was evaluated using three measures: (1) the number of compounds eliciting a significant response in the cell; (2) the noise:signal ratio (N:S ratio = response to the second-most effective stimulus/response to the most effective stimulus; Spector and Travers, 2005); and (3) entropy (Smith and Travers, 1979) calculated by the formula H = −1.43 ∑_i=1–5 _P_i_ log P_i_, where P_i_ is the proportion of the summed responses arising from a given stimulus. The first measure utilizes all responses but ignores response magnitude whereas the N:S ratio uses only two responses but takes relative magnitude into account. The entropy measure also takes response magnitude into account and utilizes all the responses. For calculating the entropy measure we substituted a very small value for zeros and responses that decreased below baseline, because the measure cannot accommodate zeros or negative numbers (Smith and Travers, 1979). When calculating the N:S ratio, we omitted responses to MSG_ai_ because, at the concentration employed (600 mm), this stimulus strongly activates sugar/umami-responsive cells and amiloride-insensitive NaCl-sensitive neurons because of the multiple constituents of the compound (i.e., Na^+^ and glutamate). Therefore, calculating N:S for this stimulus is inappropriate. Finally, we evaluated effects of activating the GABA network by deriving a threshold linear function (Semyanov et al., 2004; Atallah et al., 2012; Wilson et al., 2012; Chen et al., 2016). This analysis was conducted for both natural taste responses and for responses to light_m_ stimulation at different frequencies. To derive the function, responses of the neuron under control conditions were ordered from largest to smallest and the responses under both conditions normalized to the largest control response. The relationship between control and optogenetically-inhibited responses was then subjected to linear regression. The resulting slope captures the proportional (divisive) effect of inhibition whereas the intercept provides a measure of the subtractive effects of inhibition. A line with a slope of 1 and a *y*-intercept of 0 indicates no effect of inhibition. A purely divisive effect of inhibition changes only the slope, while a purely subtractive effect yields a line with a slope of 1 but with a *y*-offset.

#### Reconstruction of recording sites

##### Marking of recording sites

In selected instances, recording sites were marked with an electrolytic lesion made by passing current (3–8 μA for 3–10 s) at the site of recording in the NST. In other cases, lesions were made in the reticular formation or vestibular nucleus dorsal or ventral to the cell. At the end of recording, the animal was injected with a lethal dose of anesthesia (80 mg/kg ketamine and 100 mg/kg xylazine) or sodium pentobarbital (100 mg/kg) and perfused through the left ventricle with PBS, and 4% paraformaldehyde (in 0.1 m phosphate buffer) containing 1.4% L-lysine acetate and 0.2% sodium metaperiodate (McLean and Nakane, 1974). Afterwards, the brain was fixed overnight in 20% sucrose paraformaldehyde, blocked in the coronal plane and then stored in sucrose-phosphate buffer before sectioning.

##### Histology and immunohistochemistry

Coronal sections of the medulla (40–50 μm) were sectioned into two series and processed and mounted immediately or stored at –20°C in cryoprotectant until processing. One series was either left unstained to view in darkfield, stained with Weil (Berube et al., 1965) or (most frequently) black gold for delineating myelinated fibers (Schmued and Slikker, 1999). Double immunohistochemistry for P2X2 and NeuN was usually performed on the second series as described by Breza and Travers (2016). Except when noted, processing was done at room temperature and the diluent and rinsing agent was PBS. Sections were rinsed before and after treatment with 1% sodium borohydride and 0.5% H_2_O_2_. Nonspecific binding was suppressed and tissue permeabilized using “blocking serum,” a mixture of 0.3% Triton, 3% bovine serum albumin and 7.5% donkey serum, before adding the primary antibodies (anti-P2X2, 1:10,000 and anti-NeuN, 1:1000; P2X2: rabbit polyclonal antibody against the intracellular COOH terminus of the P2X2 receptor, Alomone Labs, catalog #APR003, RRID: AB_2040054; anti-NeuN RRID: AB_2040054: mouse monoclonal antibody, Millipore MAB 377, RRID: AB_2298772). After primary antibody incubation (48–72 h, 4°C), sections were thoroughly rinsed and then treated with the secondary antibody (biotinylated anti-rabbit IgG, 1:500, Jackson ImmunoResearch, RRID: AB_2337965; in blocking serum) for 1.5 h followed by an avidin-biotin mixture (Elite kit, Vector, RRID: AB_2336819) diluted in 0.1 m PB and 0.1% BSA. The chromagen reaction involved a 15-min presoak in 0.05% 3, 3-diaminobenzidine- HCl with 0.015% nickel ammonium sulfate, before adding H_2_O_2_ to achieve a concentration of 0.0015%. This reaction labeled P2X2 afferents dark brown to black. For labeling cell bodies with NeuN, we repeated the steps commencing with an anti- mouse secondary antibody incubation (biotinylated anti-mouse IgG, Jackson ImmunoResearch, RRID: AB_2338570) but did the chromagen reaction sans nickel which resulted in light brown staining of soma. Tissue sections were examined under darkfield and brightfield optics.

We located the cell in the anterior-posterior dimension based on the lesion site relative to the caudal and rostral borders of the rNST and the morphology of the section. Because lesions were large relative to the dorsoventral dimension of the taste responsive area and because our observations suggested that the cell could be located anywhere from the center of the lesion to its ventral extent (most common), we relied on protocol notes taken during the experiment as the more accurate indication of dorsoventral location. This also allowed us to reconstruct cell location in the dorsoventral axis for more cells, since only a minority were marked with a lesion at the site of recording. During recording, the transition from the overlying vestibular nucleus was typically evident as a notable decline in the amplitude of neural activity and the appearance of gustatory-driven and/or mouth-light-driven responses. We noted this transition and the depth of responses every ∼25–50 μm thereafter. The dorsal-ventral location of a neuron based on these written records was only used as the electrode passed from dorsal to ventral, since positions of electrophysiological landmarks typically shifted dorsally as the electrode was retracted.

### Behavioral studies with DREADDs

#### Mice

To study effects of activating rNST inhibitory neurons on taste-driven behavior, we used eight mice that expressed the excitatory DREADD receptor, hM3D(Gq) (Urban and Roth, 2015) and the fluorescent protein, mCherry, in rNST GAD65 neurons following viral injections (details below). We tested licking in response to different tastes in a brief-access paradigm. Five subjects were crosses between homozygous GAD65-cre males (Jax 028867; congenic version of the same line as used for the ChR2 neurophysiology crosses) and females expressing the Venus protein under the control of the VGAT promoter (RRID: ISMR_RBRC09645; Y Wang et al., 2009); three of these mice were also positive for the Venus protein. Two other subjects were the offspring of a cross between homozygous GAD65-cre mice. No difference in injection site size was apparent for mice heterozygous or homozygous for GAD65-cre. Five additional mice that were GAD65-cre X VGAT/Venus crosses (one Venus-positive) received injections of a cre-dependent AAV virus that only expressed the mCherry protein. Qualitative inspection of the injection sites in the Venus-positive mice suggested that most rNST cells expressing mCherry also expressed the Venus protein, confirming the specificity of the injections in inhibitory neurons.

#### Viral injections

An anesthetic state was induced with a cocktail of xylazine and ketamine (2.5 and 25 mg/ml, 0.06 ml/20 g bw, i.p.) and maintained with isoflurane (0.5–1%). The scalp was sanitized with alternating swipes of 70% ETOH and iodine, and a midline incision made. A small hole was drilled in the skull over the rNST and the dura removed. We made bilateral 50-nl pressure injections through glass pipettes (tip size = 40–50 μm) using a General Valve Picospritzer and monitored volume by observing the meniscus through the microscope equipped with a micrometer. rNST injection coordinates were determined by locating taste-responsive activity with a search electrode. We then replaced the electrode with the injection pipette filled with the virus and monitored neural activity through the injection pipette to assure accurate placement in the dorsal-ventral axis. The injection pipette was left in place for ∼10 min after the injection was made. Mice in the excitatory DREADD [“hM3D(Gq)/mC”] group were injected with pAAV2-hSyn-DIO-hM3D(Gq)-mCherry (ADDGENE #44361, titer: 1.8 × 10E^13^ GC/ml); mice in the mCherry viral control group (“mC”) received pAAV2-hSyn-DIO-mCherry (ADDGENE #50459, titer: 2.6 × 10E^13^ GC/ml). Mice recovered for two weeks before training. Mice were run in squads at different time points [hM3D(Gq)/mC, 2 squads; mC, 1 squad].

#### Experimental design: behavioral training and testing

Training and testing took place in a standard brief-access testing apparatus (Davis MS160-Mouse; DiLog Instruments; “Davis Rig”), in which stimulus presentation and timing was computer-controlled. Mice licked different sapid solutions from bottles fitted with sipper tubes that were automatically moved into position. A shutter controlled the mouse’s access to the bottle. For the hM3D(Gq)/mC group, the experiment proceeded in four blocks (1) training; (2) quinine testing; (3 and 4) sucrose and maltrin testing (with sucrose and maltrin order counterbalanced across two squads). Stimuli were reagent grade and dissolved in distilled water: quinine (μm: 30, 100, 1000, 3000), sucrose (mm: 30, 100, 200, 300, 600, 1000), and the maltodextin, maltrin 580 (%: 1, 2, 8, 16, 24, 32; a generous gift of the Grain Processing Corporation). Mice in the mC control group only underwent the training phase followed by testing with sucrose. For the hM3D(Gq)/mC subjects, quinine was tested before sucrose and maltrin because pilot studies suggested that mice were reluctant to sample the aversive stimulus after first experiencing the appetitive compounds. Training and quinine blocks were performed under ∼23 h of water deprivation and occurred on a daily basis with a break between the training and quinine testing. When evaluating appetitive stimuli, mice were tested under 23.5 h of food deprivation and testing was performed on alternate days.

Training began two weeks following the viral injection. During the first two training days, mice were placed in the rig for 30 min with the shutter open and a single bottle of distilled water available. On days 3 and 4, mice were permitted water access in discrete trials: a bottle moved into position, the shutter opened and remained open for 5 s after the first lick then closed for 10 s before opening again. There was no restriction on the number of trials mice could initiate in the 30-min period. Day 5 was identical to days 3 and 4, with the addition of a clozapine-N-oxide (CNO) injection to check for any untoward effects. This same 5-s trial structure was used for the taste stimulus testing. Before each taste test block, the relevant stimulus was introduced in a single session without any injections. Subsequently, a given stimulus was tested four times, twice with saline injections (∼0.1 ml/10 g bw, i.p.) and twice with CNO (1.0 mg/kg, in saline, i.p.). Injections were given 30 min before the test session. Injection order was counterbalanced across mice. For the hM3d(Gq)/mC groups, an additional 30 min water trial session with a CNO injection was given between the quinine and the sucrose and maltrin testing blocks and then again just before perfusion to assess the possible development of effects on lick rate over time. We found that under water deprivation the modal lick rate was initially unaffected by the CNO injection but that it did slow by the end of all testing [mean modal interlick interval (ILI), *N* = 8 mice: before taste testing: saline = 115.5 ms, CNO#1 = 113.6 ms; after quinine testing: CNO#2 = 113.5 ms; after all testing, CNO#3 = 141.6 ms (ANOVA: *p *=* *0.0000087; *post hoc* Bonferroni-adjusted *t* tests: saline vs CNO#1 and CNO#2, *p =* 1.0; saline vs CNO#3, *p *=* *0.009]. Thus, we compensated for lick rate changes over time using a standardized lick ratio (see below). Following the last test period, mice were perfused as in the neurophysiological experiments and brains cut into 40-μm sections. Sections were mounted onto slides and fluorescent photomicrographs taken of the injection site. All injections were centered in rNST and mainly confined to the nucleus.

#### Statistical analysis

Mice were omitted from analysis if they sampled each stimulus on fewer than two trials per session; this yielded final *N*s of 7 mice each for quinine and sucrose, and 6 for maltrin for the DREADD-injected mice. There were five mice in the mC control group. Responses to quinine were quantified as the ratio of the number of licks to quinine relative to the average number of licks to water for a given test day. For sucrose and maltrin, responses were quantified as a standardized lick ratio (Glendinning et al., 2002) where we normalized the number of licks relative to the maximum possible licks based on the modal ILI for a given mouse and session. These standardized measures controlled for the changes in lick rate that occurred in the sucrose and maltrin testing phases (see above). Analyses were performed as repeated measures ANOVAs with concentration and drug state as the independent variables. These were followed by Bonferroni-adjusted paired *t* tests for each concentration. Statistics were performed in Systat (v13), Excel (2016), and GraphPad Prism (v9.1.1). The significance level was set at *p *< 0.05. Fits of the average curves were made using nonlinear regression with the equation:

$$Y\;=\;Minimum\;Response\;+\;\left(Maximum\;Response-Minimum\;Response\right)/(1+10^{\left(\left(LogEC50-X\right)*Hill\;Slope\right)}),\text{where Y is the standardized lick ratio and X is concentration}.$$

## Results

### Putative GABA Neurons (*N* = 38)

Thirty-eight cells were classified as putative GABA neurons because they responded faithfully and at short latency to light pulses delivered through the optrode. Fifteen responded to taste (G+_TASTE_) and four to oral somatosensory stimulation or depressing the mandible (G+_MECH_, two of these were slightly lateral or ventral to the NST borders). An additional 19 neurons failed to respond to either taste stimulation or fluid flow (G+_UNR_) and a lack of responsiveness to stroking the oral mucosa was further confirmed for nine of these cells. Quantitative data on evoked responses to 10-Hz light stimulation were obtained for 31 light-responsive neurons and for 26 cells over an extended frequency range. The remaining seven light-entrained neurons were cells with obvious synchronized responses to light pulses and histologically verified to be in the NST but with isolation insufficient for accurate quantification (*N* = 4) or for which only protocol notes were taken (*N* = 3); these cells were used only in the histologic analyses.

**Figure 2.**
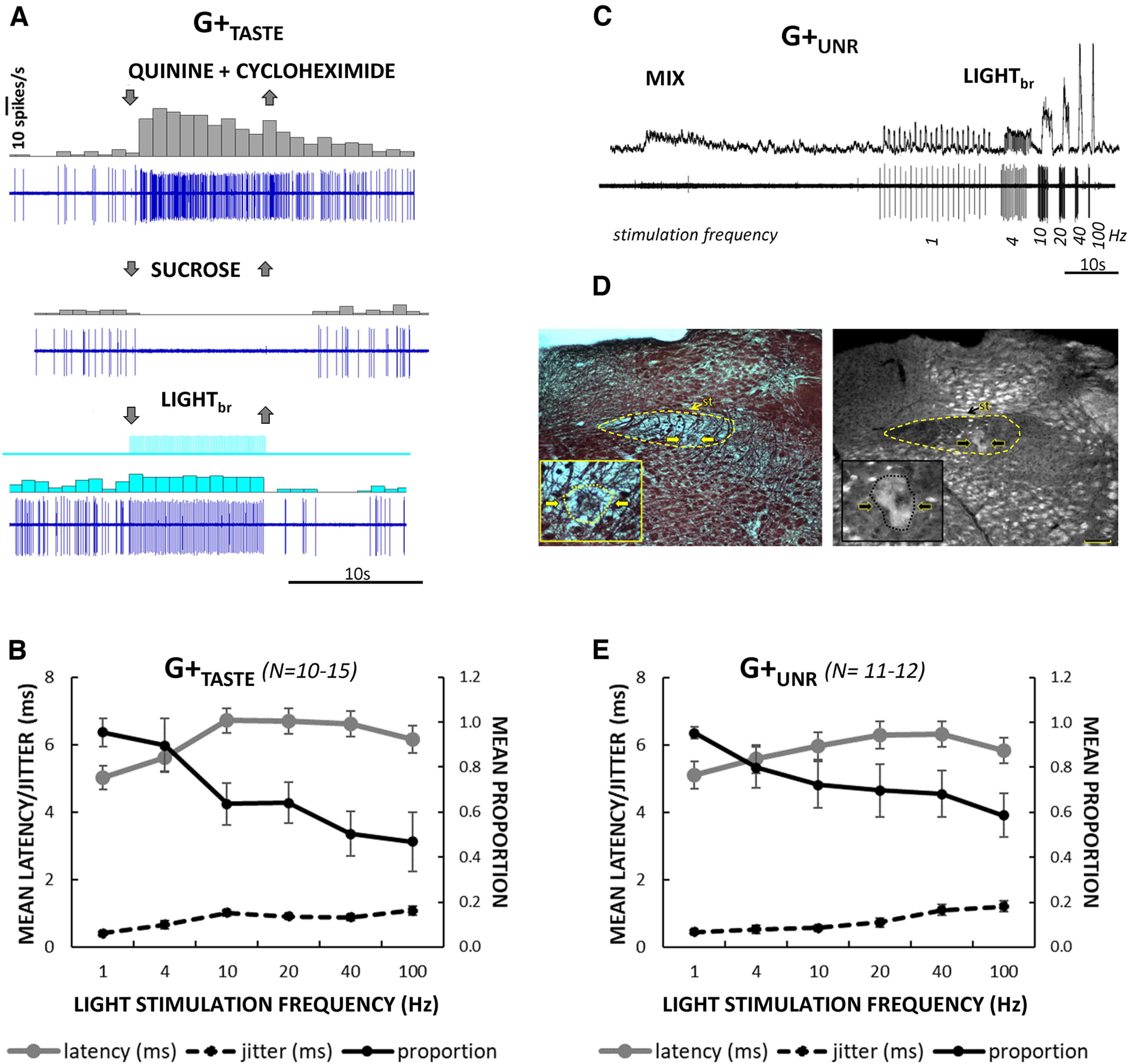
Some rNST putative inhibitory neurons are gustatory-responsive (G+_TASTE_), but others are unresponsive to orosensory stimulation (G+_UNR_). ***A***, Response characteristics of an exemplar G+_TASTE_ neuron. Raw record and peri-stimulus time histograms (1-s bins) for a G+_TASTE_ neuron for the bitter mixture, sucrose and light_br_. At 10-Hz stimulation, this neuron responded reliably to light pulses (100%, 100 trials) with short latency and low jitter (5.9 ± 0.72 ms). ***B***, Average (±SEM) latency, jitter, and proportion of trials followed across the population of G+_TASTE_ cells (*N* = 15 for 10 Hz; *N* = 10–11, other frequencies). ***C***, ***D***, Example of a G+_UNR_ neuron. ***C***, Integrated (top) and raw (bottom) records showing recording from a nonspontaneously active neuron driven by light_br_ but unresponsive to taste stimulation. Note the small background taste response evident in the integrated record. The cell followed 10-Hz light_br_ pulses reliably and at short latency and low jitter (88%, 100 trials, 5.8 ± 0.68 ms). ***D***, *Post hoc* histology for the cell in panel ***C*** (left, brightfield image of black-gold staining; right, darkfield image from an adjacent unstained section). Dashed lines indicate the approximate border of the nucleus. Arrows point to the medial and lateral sides of the lesion in each panel. Insets show magnified views of the lesion outlined with dotted lines. The lesion in the black-gold section appears as a subtle disruption of staining with a coagulated center zone; in the darkfield image, the lesion appears as a light region surrounding a central dark zone. Lesions indicate that the cell was in the ventral region of the nucleus, corresponding to the ventral subdivision (Whitehead, 1986; Ganchrow et al., 2014). Scale bar: 100 μm; medial is to the left. st, solitary tract. ***E***, Average (±SEM) latency, jitter, and proportion of trials followed, across the population of G+_UNR_ cells for which we tested an extended frequency range (*N* = 12, 10 Hz, 11 other frequencies).

Figure 2 shows examples of neurons classified as putative taste G+_TASTE_ (Fig. 2*A*) or unresponsive G+_UNR_ (Fig. 2*C*,*D*) inhibitory cells based on their reliable short-latency, low-jitter responses to brain light and the presence or absence of a taste response. The G+_TASTE_ cell responded most robustly to the bitter mixture and well to NaCl (data not shown) but, somewhat atypically, was silenced by sucrose stimulation. In addition, 10-Hz light stimulation evoked an entrained response. Figure 2*B* shows the mean latency, jitter, and following for light_br_ stimulation for the G+_TASTE_ neurons across a range of frequencies. An example of a G+_UNR_ neuron is shown in Figure 2*C*,*D*. This neuron lacked spontaneous activity and a taste response. Neither did it respond to fluid flow, stroking the oral mucosa, or depressing the mandible (data not shown). Indeed, this cell was detected only because of its synchronous response to light_br_ stimulation as the electrode passed to the ventral region of the nucleus, likely in the ventral subdivision (Whitehead, 1986; Ganchrow et al., 2014), (Fig. 2*D*) where taste activity was waning. Figure 2*E* shows the mean light-evoked latency, jitter, and following for the population of G+_UNR_ neurons as a function of stimulus frequency. Four additional neurons responsive only to oral somatosensory stimulation (G^+^_MECH_) responded to light with similar latency, jitter, and following characteristics. Across all putative inhibitory cells (11 G+_TASTE_, 11 G+_UNR_, 4 G+_MECH_), for which data for 1-Hz stimulation was available, light evoked responses on 95.9 ± 1.6% of trials. Thus, like “optotagged” cells originally described by Lima in auditory cortex (Lima et al., 2009) highly faithful following was apparent at 1 Hz.

**Figure 3.**
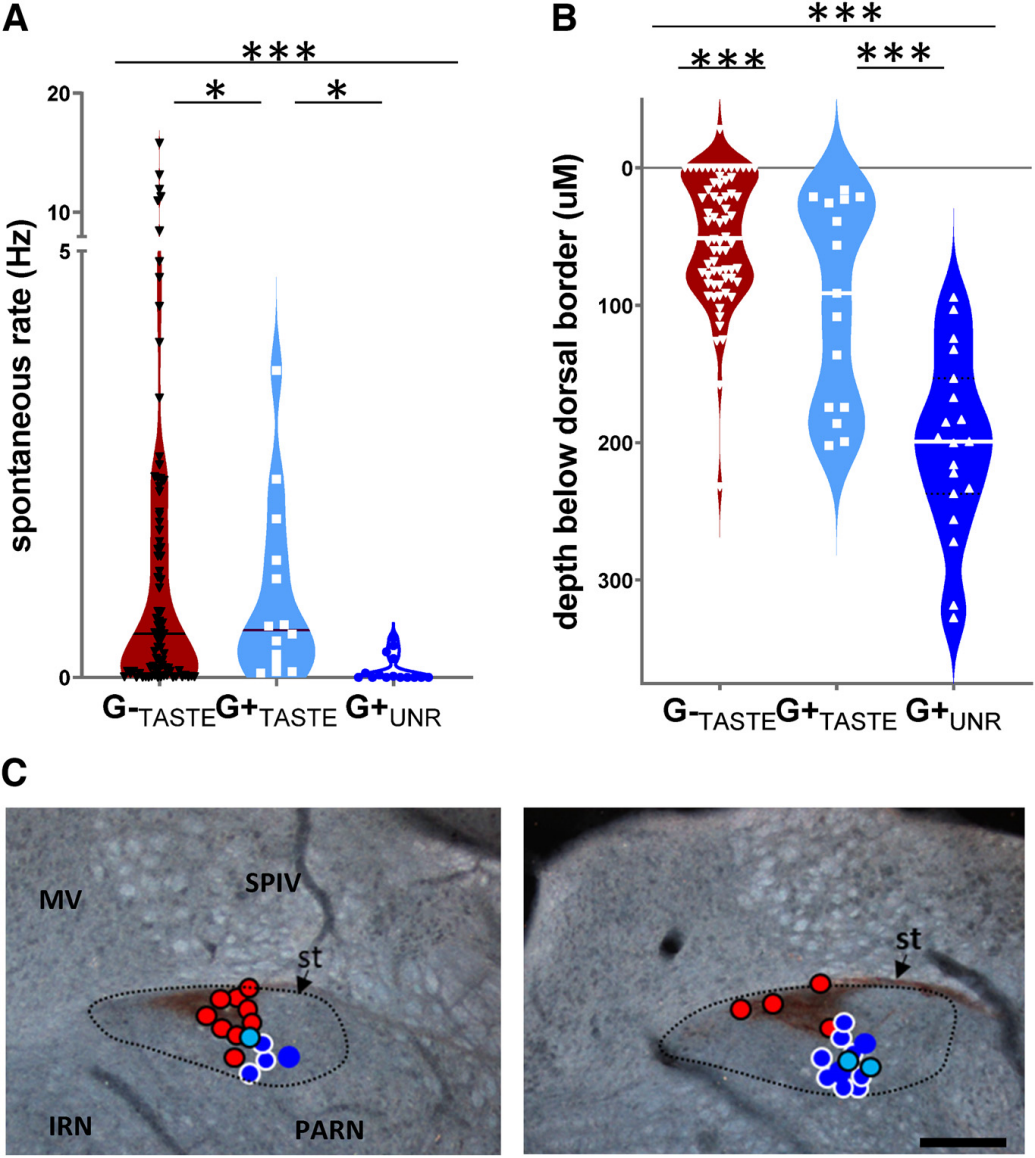
G-_TASTE_, G+_TASTE_, and G+_UNR_ neurons have distinctive distributions of spontaneous rate and locations in the dorsoventral axis. ***A***, Violin plots depicting spontaneous rate of individual G-_TASTE_, G+_TASTE_, and G+_UNR_ neurons. Horizontal lines indicate medians. A Kruskal–Wallis ANOVA (*N* = 113) indicated significant differences between all three cell types (***G-_TASTE_ vs G+_TASTE_, *p =* 1.66e-06; G-_TASTE_ vs G+_UNR_, *p = *1.56e-06, G+_TASTE_ vs G+_UNR_, *p =* 1.13e-05). ***B***, Depth of individual G-_TASTE_, G+_TASTE_, and G+_UNR_ neurons relative to the dorsal border of the NST identified electrophysiologically by the transition from high-amplitude activity characteristic of the vestibular nuclei to lower-amplitude activity responsive to gustatory and oral somatosensory stimulation. Horizontal lines indicate medians. ANOVA indicated a significant difference by type (*p =* 1.75e-11, ANOVA, *N* = 102) and Tukey’s *post hoc* tests indicated significant differences *** between all three cell types (G-_TASTE_ vs G+_TASTE_, *p *= 0.008; G-_TASTE_ vs G+_UNR_, *p =* 1.07e-05, G+_TASTE_ vs G+_UNR_, *p =* 1.17e-05). ***C***, Locations of the subset of neurons marked by a lesion made at the site of recording; symbols are superimposed on darkfield photomicrographs of NST sections from more rostral (left) and caudal (right) levels of the rNST immunostained for P2X2 gustatory afferent fibers which appear as the wedge-shaped dark brown areas. Symbol colors are the same as for panel ***A***, but outlines were added to enhance visibility: G-_TASTE_ neurons, red circles/black borders; G^+^_TASTE_ neurons, light blue circles/black borders; G+_UNR_ neurons, dark blue circles/white borders. A few G+_MECH_ neurons are also plotted: blue circles/no outlines. Dotted outline indicates the approximate borders of the nucleus. IRN, intermediate reticular nucleus; PARN, parvicelluar reticular nucleus; MV, medial vestibular nucleus; SPIV, spinal vestibular nucleus; st, solitary tract. Scale bar: 200 μm.

Putative GABA and non-GABA neurons differed based on characteristics other than their light-synchronized responses (Fig. 3). The mean spontaneous rate was highest in the G-_TASTE_ cells and lowest in G+_UNR_ neurons (Fig. 3*A*). Indeed, whereas most G-_TASTE_ (94%) and all G+_TASTE_ neurons exhibited some level of resting activity, only 46% of G+_UNR_ neurons were observed to fire any spikes in the absence of light_br_ stimulation. In addition, based on microdrive coordinates, G-_TASTE_, G+_TASTE_, and G+_UNR_ neurons were preferentially staggered from dorsal to ventral in the nucleus (Fig. 3*B*). This finding was consistent with what we observed after reconstructing the histology for the subset of cells for which we made lesions at the site of recording (Fig. 3*C*).

**Figure 4.**
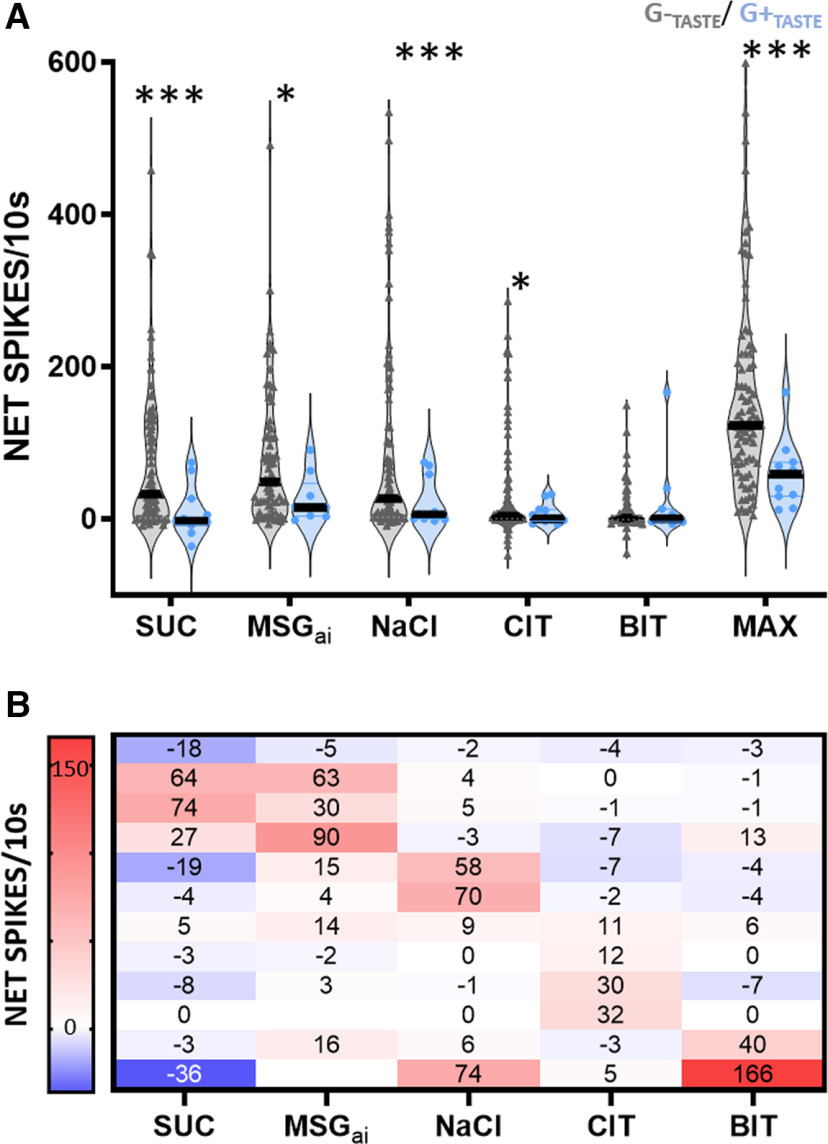
G-_TASTE_ and G+_TASTE_ neurons respond to the same taste qualities, but responses are weaker in G+_TASTE_ cells. ***A***, Violin plots showing the individual (symbols) and median (lines) responses to five standard taste stimuli and for the maximum response (MAX) for a given cell for G-_TASTE_ (*N* = 84 except for BIT, *N* = 82 and MSG_ai_, *N* = 72) and G+_TASTE_ (*N* = 11, except for MSG_ai_, *N* = 9; 1 G+_TASTE_ neuron that only responded in an inhibitory fashion was omitted from these means but appears in the individual responses in panel ***B***). An ANOVA (excluding MSG_ai_) showed a main effect for cell type: *p *= 0.009, but not stimulus: *p *= 0.118, or an interaction between stimulus and cell type (*p *= 0.147). *Post hoc* Bonferroni-adjusted *t* tests with Welsh’s correction for unequal sample sizes indicated that responses were significantly smaller for all stimuli except BIT (***SUC: *p *= 0.0005, MSG_ai_: *p = 0.0085, NaCl: ***p = 0.0005, CIT: *p = 0.013). The lack of a significant effect for BIT remained when the neuron with the highest firing rate for this stimulus was removed from the analysis. The maximum response was also smaller (***p < 0.0006). ***B***, Heat map with net responses showing individual response profiles for each G+_TASTE_ neuron.

Figure 4 summarizes the gustatory response characteristics of the population of putative inhibitory G+_TASTE_ cells that we were able to test with at least four of the 5 standard taste qualities (*N* = 12) compared with “G-_TASTE_” (*N* = 84). Stimuli representing each quality drove responses in both G+_TASTE_ and G-_TASTE_ cells (Fig. 4*A*). Because there was only a small sample of G+_TASTE_ neurons, we did not consider it meaningful to compare response profiles of G+_TASTE_ and G-_TASTE_ neurons in detail. Thus, we did not use cluster analysis to divide them into chemosensitive groups or evaluate their breadth of tuning. However, as reflected in the overall mean responses, neurons optimally responsive to each taste quality were evident (Fig. 4*B*), as was the case for G-_TASTE_ cells (see below). Despite these broad similarities, a salient characteristic of G+_TASTE_ neurons was that, with one exception, each taste stimulus elicited a markedly and significantly smaller response than in G-_TASTE_ cells. Indeed, even when the maximal response (MAX) for a given neuron was considered, the average taste-driven response of G+_TASTE_ cells was only 37% as great as in G-_TASTE_ neurons. The exception to the trend for all stimuli to evoke a smaller response was for the bitter stimulus, but because of the low rates of bitter responsiveness in the G-_TASTE_ cells in our sample and the single G+_TASTE_ neuron that had a robust bitter response, this observation must be considered with caution.

**Figure 5.**
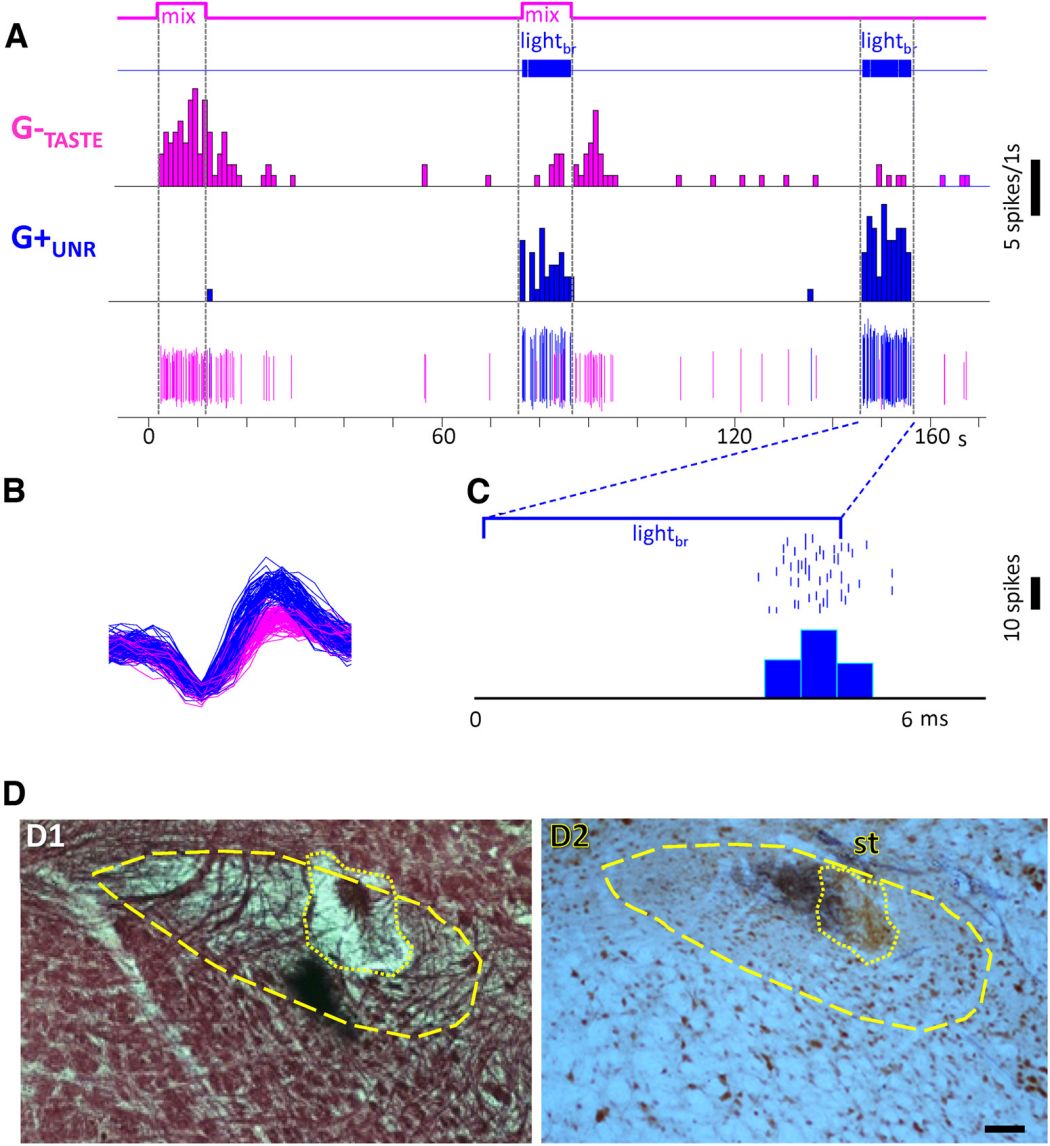
Some putative inhibitory rNST neurons are unresponsive to taste stimulation and intermixed with non-GABA taste responsive neurons. ***A***, Peristimulus time histogram and windowed spikes showing a neuron unresponsive to gustatory stimulation but driven by light_br_ (i.e., a G+_UNR_ neuron, windowed in blue) recorded simultaneously with a (marginally isolated) taste-responsive neuron where light_br_ suppressed taste-elicited activity (i.e., a G+_TASTE_ neuron, windowed in pink). ***B***, Expanded time base showing the waveform of the windowed cells. ***C***, Light_br_-triggered histogram (0.5-ms bins; light onset at time = 0) showing that the G+_UNR_ cell responded at a short latency (5.89 ms) with low jitter (0.42 ms) on most (76/100 at 10 Hz) trials. ***D***, Photomicrographs illustrating the lesion at the recording site. ***D1***, Staining with black-gold showing myelinated fibers. ***D2***, Alternate sections were double immunostained for NeuN (brown) and P2X2 (black). Approximate borders of the rNST are shown in dashed lines; lesions are outlined with dotted lines. In NeuN-stained sections, lesions were often evident based on homogeneous instead of cellular staining, presumably an immune reaction against damaged tissue because of the anti-mouse secondary antibody. Scale bar: 100 μm. st, solitary tract.

Although G-_TASTE_ and G+_TASTE_ neurons were differentially distributed in the nucleus, there was clear intermingling between the various cell types. Figure 5 shows an example where a light-driven G+_UNR_ neuron (larger potentials; blue wavemark) was recorded simultaneously with a G-_TASTE_ cell (small pink wavemark; only used for illustrative purposes). The G-_TASTE_ cell responded robustly to a mixture of the four classic taste stimuli (Fig, 5*A*, mix) and to sucrose and MSG_ai_; NaCl, citric acid and the bitter mixture were ineffective. Moreover, whereas activity in the G+_UNR_ neuron was synchronized to light_br_, the taste-elicited activity in the G-_TASTE_ neuron was suppressed (Fig. 5*A*, mix + light_br_). The remainder of the paper considers such suppressive effects of activating the rNST GABA network on the taste responses of putative non-GABA cells.

### Effects of the NST GABA Network on taste responses in non-GABA (G-taste) cells (*N* = 54)

#### Population effects

**Figure 6.**
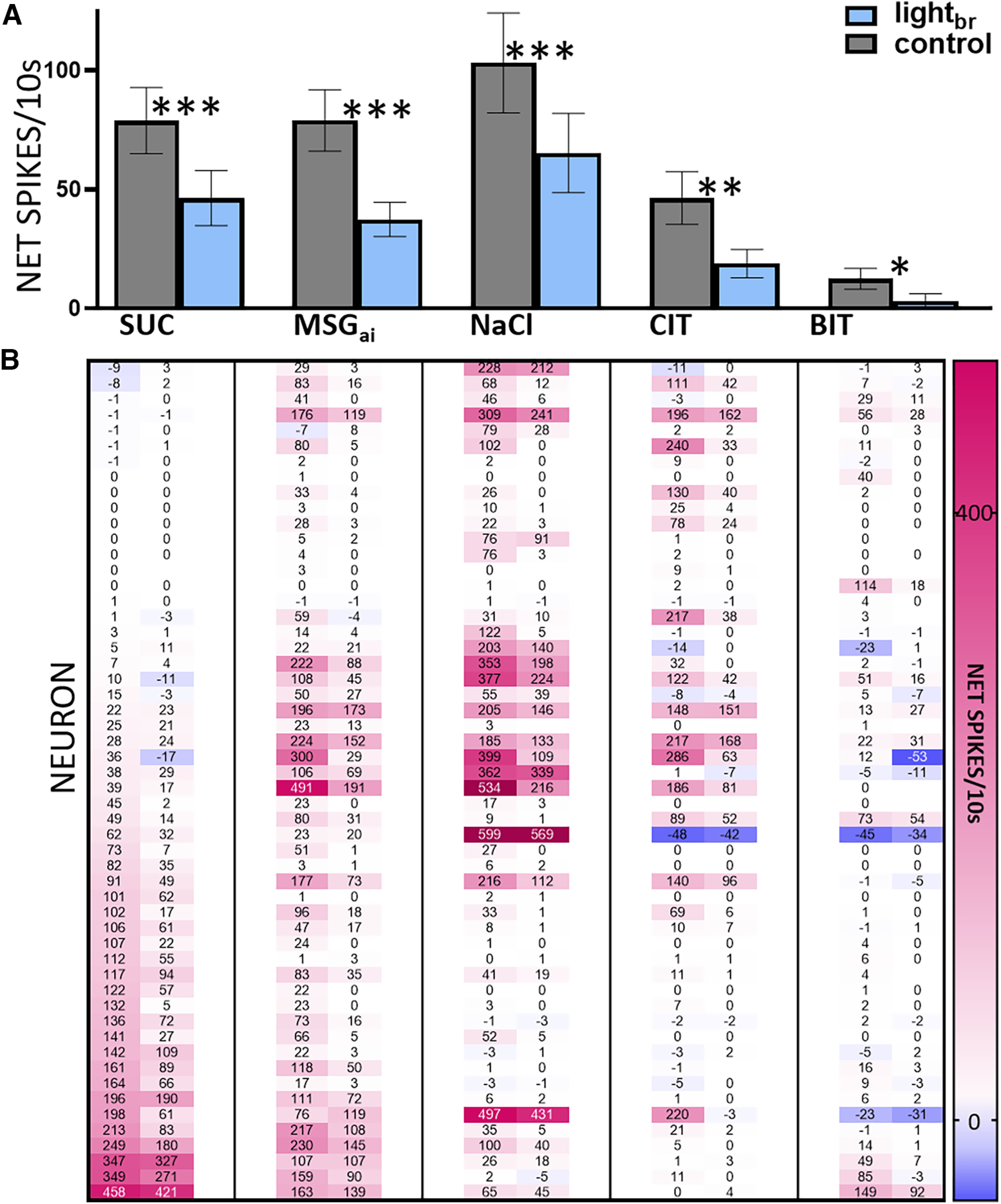
Responses to all taste qualities are suppressed by optogenetic stimulation of the GABA network. ***A***, Mean net (±SEM) responses to each stimulus for 54 G-_TASTE_ neurons tested with stimulus set A. A repeated-measures ANOVA showed main effects of light (*p = *5.11e-08) and stimulus (*p = *5.37e-05). (Because there were a few missing data points for the brain-light stimulated condition for stimuli ineffective under control conditions, the sample size for the ANOVA was reduced to 46.) There was also a light X stimulus interaction (*p *= 0.004), but this was not indicative of differential effects of inhibition across stimuli: brain light suppressed responses for each stimulus (Bonferroni-adjusted *t* tests: SUC: ***p =7.75e-07, *N* = 54; MSG_ai_: ***p = 1.92e-05, *N* = 53; NaCl: ****p = 1.53e-04, *N* = 51, CIT: **p = 0.005, *N* = 50, *BIT: *p *= 0.015, *N* = 46). ***B***, Corresponding heat map showing individual responses of each neuron and stimulus under light-stimulated and control conditions, lined up with the means in the panel ***A***. Cells were ordered by their responsiveness to sucrose with no light. Response is color-coded according to the legend on the right and indicated by the numbers in a given rectangle; rectangles without numbers indicate missing data points.

Across the 84 non-GABA taste cells described in the preceding section, 54 were tested with stimulus set A (Table 1) and used to analyze the effect of activating the rNST GABA network on a population which likely includes excitatory projection neurons. Optogenetic activation of the rNST GABA network substantially and significantly suppressed mean responses to each stimulus (Fig. 6*A*), suggesting that entire gustatory spectrum is susceptible to inhibitory influences. The consistency of this effect across individual neurons and qualities is apparent in the heat map (Fig. 6*B*). Although the magnitude of suppression varied and a few neurons were unaffected, both cells with large and small responses were vulnerable to suppression. In addition, responses to the “best” and “sideband” stimuli in a given cell were all susceptible to inhibition. The firing rate during sustained flow of AS likewise declined during light stimulation (11.8 ± 3.1 vs 7.5 ± 2.2 spikes/10 s, *p *= 0.001, *N* = 54).

#### Relative response profiles and ensemble coding remain stable during GABA activation

**Figure 7.**
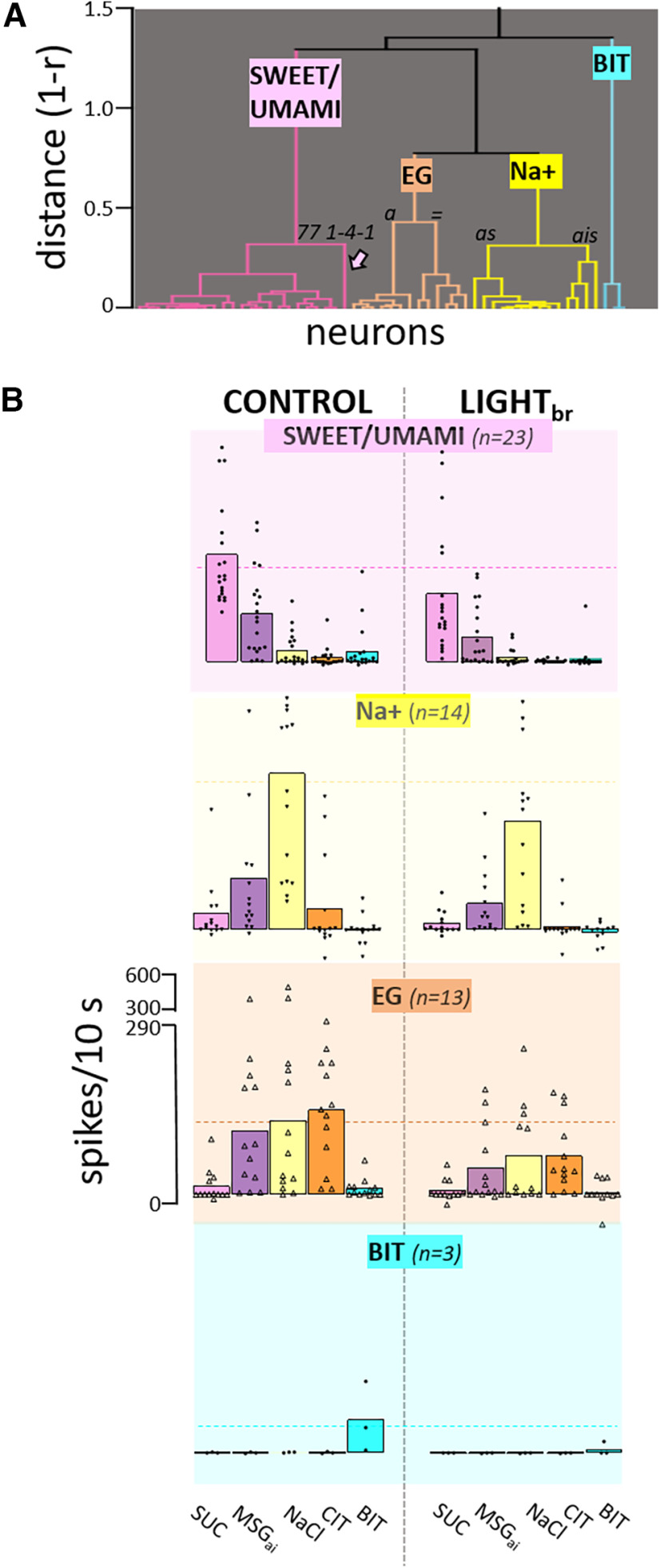
Activation of the GABA network suppresses responses in all chemosensitive clusters but the shape of the profiles is largely unchanged. ***A***, Cluster tree showing segregation into 4 main chemosensitive groups. One neuron was omitted from the clustering process because it only joined the rest of the neurons at a distance (1-r) of 0.898. The resulting clusters largely reflected the stimulus or stimuli evoking the optimal response(s) in a cell: sucrose/MSG_ai_ (“SWEET/UMAMI”), NaCl (“Na^+^”), electrolyte generalists (“EG,” comparable mean responses to MSG_ai_, NaCl, and CIT), and a small group responding nearly exclusively to the quinine/cycloheximide cocktail (“BIT”). We used these four groups for further analysis to be parsimonious and to maintain adequate sample sizes. However, there was heterogeneity within the Na^+^ and EG groups: the Na^+^ group was mostly comprised of cells with optimal responses to NaCl and minimal MSG_ai_ responses (*as*, amiloride sensitive, *N* = 10). A smaller group of Na^+^ cells (*ais*, amiloride insensitive, *N* = 4) responded optimally to NaCl but had robust responses to MSG_ai_. The EG group split into neurons that responded similarly to MSG_ai_, NaCl and CIT (*N* = 6, “=”) and those with a more dominant response to CIT (*N* = 7, “*a*”). The arrow indicates the last neuron that joined the “SWEET/UMAMI” cluster and is referred to in Figure 8. ***B***, Mean response profiles (and individual data points) for each cluster under control conditions and during activation of the GABA network by light_br_. Activating the GABA network had a potent effect on response magnitude but the relative order of effectiveness of the stimuli is virtually identical under the two conditions. Mixed ANOVA (excluding the BIT cluster), main effects-cluster: *p *= 0.545; light_br_: *p =* 6.1 e-08, and stimulus: *p =*3.38e-11, no interaction between cluster and light_br:_ *p *= 0.251. There was an interaction between stimulus and cluster (*p =*9.99e-16), suggesting that the neuron groups were distinct. The sample size for the ANOVA was reduced to 42 because of a few missing data points for the light condition for stimuli that did not evoke a response under control conditions. When the ANOVA was repeated with the full data set by substituting missing values with the control (null) values, conclusions were identical.

Inhibitory effects for all qualities were likewise apparent when neurons were segregated according to chemosensitive profile. A cluster analysis of control responses suggested four main groups principally defined by the quality(ies) associated with the standard taste stimulus or stimuli that elicited the largest response(s) (Fig. 7*A*). Although the cluster tree reveals heterogeneity within groups, the average response profiles for the clusters were distinctive. Neurons responding optimally to sucrose also responded robustly to MSG_ai_, but poorly to other qualities and thus were labeled “SWEET/UMAMI” cells. Another chemosensitive cluster (“Na^+^”, *N* = 14) responded 3× as well to NaCl as any other stimulus including MSG_ai_, despite its higher sodium content, presumably reflecting attenuation by the amiloride in the MSG_ai_ cocktail. A third group responded nearly equivalently to NaCl, citric acid and MSG_ai_, and thus was considered an electrolyte generalist group (“EG,” *N* = 13). The very small number of remaining neurons were characterized by responding optimally and nearly exclusively to the bitter mixture (“BIT”, *N* = 3). ANOVA indicated significant main effects of light and stimulus and an interaction between neuron group and stimulus but no interaction between light and neuron group, suggesting that the different chemosensitive types were similarly susceptible to inhibition (see figure caption for details). To probe this further, we analyzed the degree of absolute and proportional suppression for the classic taste stimulus eliciting the largest response in each neuron; again, there was no impact of neuron group on this measure (ANOVAs, *p*s > 0.1). These ANOVA analyses were performed without the BIT cells because of the small sample size, but it is worth pointing out the activity in each of the three cells tested was suppressed by optogenetic inhibition, suggesting that such cells are also susceptible to inhibition.

**Figure 8.**
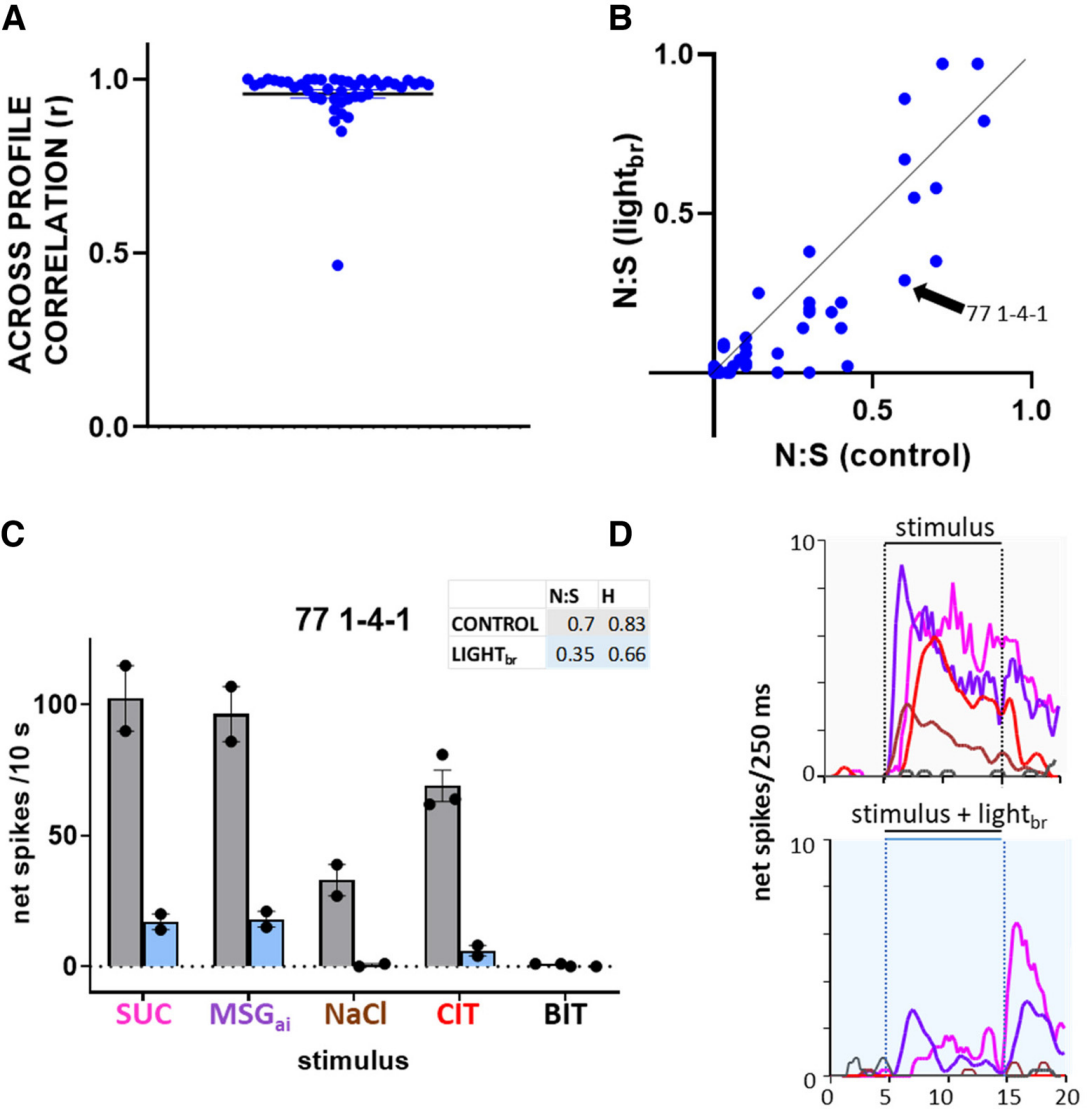
Response profiles are highly similar but modestly narrower under GABA activation. ***A***, Across-profile correlations (Pearson’s *r*) for individual neurons during control conditions and during light_br_. Dots show results for individual cells; horizontal lines indicate mean and SEM. ***B***, Scatterplot depicting the N:S ratio during control conditions and during light_br_. Points on the solid line indicate a neuron with the same N:S ratio under the two conditions. Cells below the line have narrower tuning and those above have broader profiles with light. The arrow points to cell 77 1-4-1, illustrated in ***C***, ***D*** that shows an example of sharpened tuning under GABA activation. ***C***, Under control conditions, neuron 77 1-4-1 was very broadly tuned (Fig. 7 shows that it was the last cell to join the SWEET/UMAMI cluster). This cell responded significantly to 4/5 qualities, reflected in high N:S and entropy measures. During light_br_, responses were greatly depressed and only SUC and MSG_ai_ remained effective. Moreover, both the N:S and entropy measures declined. Bars indicate mean 10-s net responses to the five stimuli; dots show individual replications; the vertical line indicates the SEM. ***D***, Average time course (250-ms bins) of the responses. X axis indicates time in s. Responses are color coded as in the labels panel ***C***. Interestingly, responses to MSG_ai_ and NaCl occurred at a shorter latency than SUC and CIT and the relative time course of SUC and MSG_ai_ remained unchanged under light_br_. Because responses to stimulation of both the anterior and posterior mouth were recorded on this track, we speculate that the latency differences may be because of differences in the receptive fields for these two stimuli.

Importantly, though mean responses were clearly smaller during optogenetic inhibition, Figure 7*B* shows that the shapes of the average chemosensitive profiles were highly similar during GABA activation. The average order of effectiveness across stimuli was virtually identical under either condition. Ten cells (three SWEET/UMAMI, two Na^+^, three EG, and two BIT) became entirely unresponsive during optogenetic stimulation but the stimulus eliciting the largest response was unaltered for 40 of the 44 remaining neurons. Indeed, across individual cells, the correlation between response profiles for the two conditions was very high (mean *r* = +0.96 ± 0.13, *N* = 44; Fig. 8*A*), with all but one cell exhibiting a correlation greater than +0.85.

Despite this high degree of correlation, neurons did become more narrowly tuned during optogenetic inhibition. These effects were statistically significant, albeit modest. For cells that remained responsive during light_br_ stimulation, we assessed tuning using three measures: (1) the number of responses meeting criteria; (2) the entropy measure; and (3) the N:S ratio. With each measure tuning became sharper during light_br_ stimulation. Under control conditions, the average number of responses in a cell was 2.8 ± 0.2 which decreased to 2.1 ± 0.2 during optogenetic stimulation (*p *= 4.54e-05, *N* = 44). Likewise, entropy (H) declined from 0.537 ± 0.031 to 0.437 ± 0.039 (*p *=* *4.53e-05, *N* = 44) and the N:S ratio changed from 0.27 ± 0.04 to 0.20 ± 0.04 (Fig. 8*B*, *p *= 0.002). An ANOVA incorporating chemosensitive group as a factor showed main effects of light_br_ for each measure (N:S, *p *=* *0.003; number of responses: *p *=* *5.14e-5; entropy, *p *=* *2.61e-04) and a main effect of cluster for both entropy (*p *= 0.023) and N:S (*p = *7.35e-05), but no interaction between chemosensitive group and light_br_ for any measure (N:S, *p *=* *0.91; number of responses: *p = *0.61; entropy, *p* = 0.33), suggesting a common effect in narrowing tuning across chemosensitive clusters. Figure 8*C*,*D* illustrates one of the more prominent effects of inhibition on tuning that occurred in a particularly broadly-tuned SWEET/UMAMI cell.

**Figure 9.**
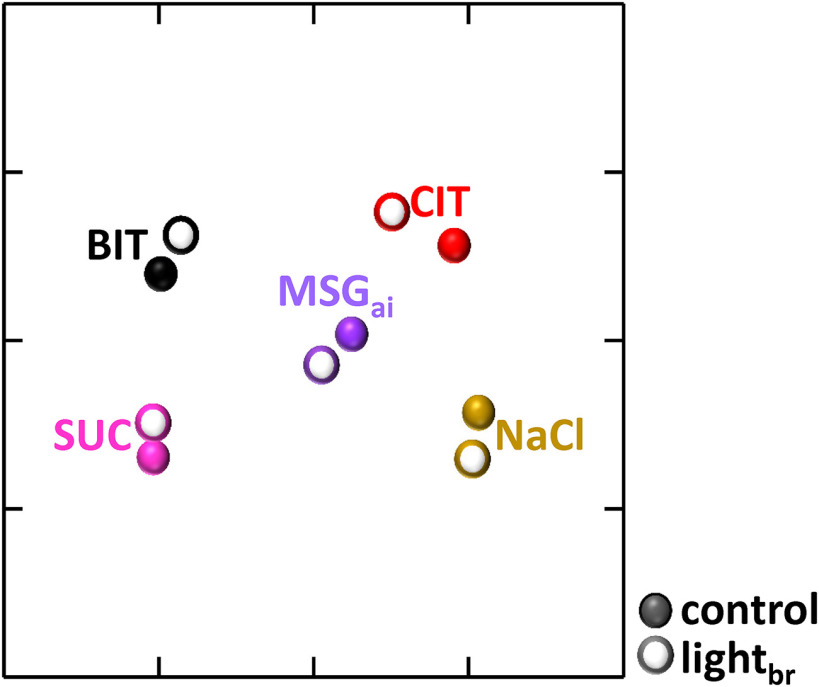
Ensemble patterns remain stable during GABA network activation. Multidimensional scaling plot of the five stimuli under control conditions (closed symbols) and during optogenetic activation of the GABA network (open symbols). The placement of the stimuli is highly similar under the two conditions.

To assess whether these changes in tuning affected ensemble coding, we calculated across-neuron correlations and depicted them in a multidimensional scaling plot (Fig. 9). Relationships among stimuli were strikingly similar for control responses and responses suppressed by stimulation of the GABA network. Although across-neuron correlations did exhibit some subtle shifts during optogenetic stimulation, these shifts were not sufficient to alter the overall pattern. Moreover, a given stimulus was positioned very closely in the taste space during the control and optogenetic stimulation conditions and correlations between these pairs were high (*r* = 0.72–0.93), exceeding correlations between any other pair of stimuli. Thus, we conclude that the main effect of nonselectively activating the rNST GABA network is to change response gain; in other words, the effects of inhibition are largely divisive rather than subtractive (Semyanov et al., 2004; Atallah et al., 2012; Wilson et al., 2012; Chen et al., 2016).

#### Effects of GABA network activation on mouth-light driven responses

**Figure 10.**
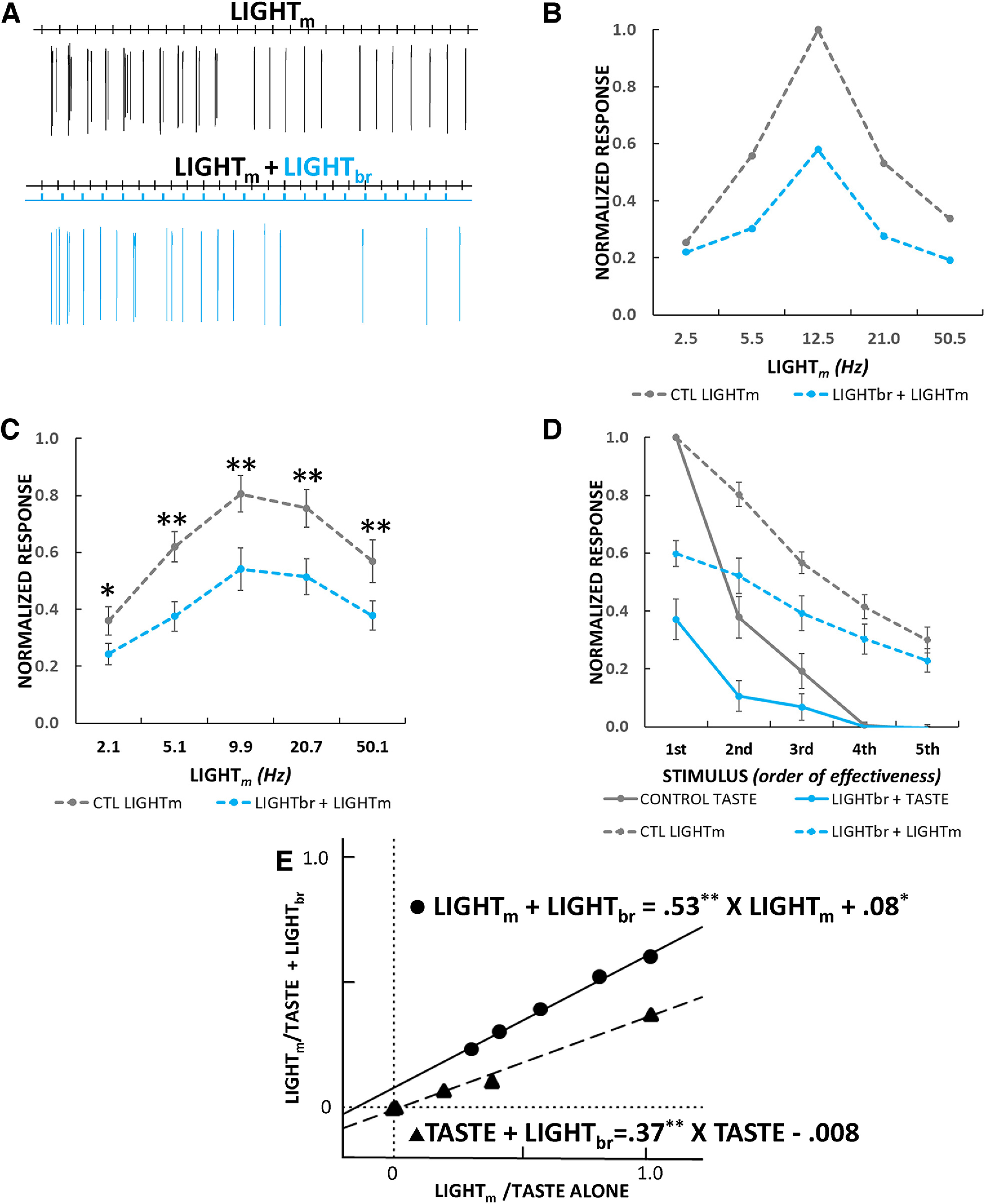
Threshold linear analysis of taste and light_m_ responses suggests a larger divisive than subtractive effect. ***A***, Firing pattern of an individual neuron to repetitive light_m_ stimulation (12.5 Hz, 5 ms, 10 mW) under control conditions and during activation of the GABA network with light_br_ (10 Hz, 5 ms). Light_m_ stimulation elicited time-locked firing that was suppressed by light_br_ stimulation. ***B***, Responses from the cell shown in ***A*** at different stimulation frequencies. ***C***, Average normalized response rates (*N* =13–16) from stimulation at ∼2–50 Hz under control conditions and their suppression during light_br_. ANOVA: light_br_, *p = *2.84e-06, light_br_ frequency, *p *= 0.041, light_br_ X light_br_ frequency, *p *= 0.857. Bonferroni-adjusted *t* tests: 2 Hz, *p *= 0.015 (*N* = 13); 5 Hz, *p *= 0.005 (*N* = 16); 10 Hz, *p = *1.18e-03 (*N* = 16); 20 Hz, *p = *2.11e-05 (*N* = 16); 50 Hz, *p *= 0.005 (*N* = 15). ***D***, “Tuning curves” for same neurons as in ***C*** for light_m_ and taste responses derived by ordering normalized responses from largest to smallest under control conditions and then ordering stimuli during light_br_ identically. ***E***, Linear regression of control versus light_br_ stimulation for taste and light_m_ responses. In both cases, activating the GABA network had a larger effect on slope than on the Y intercept, suggesting a more prominent divisive than subtractive effect.

One limitation of the conclusion that activation of the inhibitory network mostly affects gain is that the present sample was dominated by SWEET/UMAMI and Na^+^ (amiloride-sensitive) neurons, types that remained narrowly tuned despite attempts to broaden profiles by using relatively high stimulus concentrations. To overcome this limitation, we used a second approach to activate cells over a more controlled range of firing rates by stimulating taste bud cells expressing ChR2 with blue light pulses directed to the mouth at different stimulation frequencies. We used this approach in a subset of G-_TASTE_ neurons when the cell remained well-isolated following taste stimulation. Indeed, a particularly high quality of isolation was necessary for the mouth-light protocol, since this stimulus often recruited additional small potentials that interfered with accurate quantification of light_m_-driven responses. Nevertheless, we were able to assess such responses in 16 neurons including seven SWEET/UMAMI, four Na^+^, and five EG neurons. An example of optogenetic GAD65 inhibition of the response of a single neuron to mouth-light (light_m_) stimulation is shown Figure 10*A*,*B*. Figure 10*C* illustrates the mean responses from all 16 cells, normalized to the maximum control response to a given neuron. Mean responses to light_m_ stimulation increased from ∼2–10 Hz but at higher stimulation frequencies responses saturated and then declined; optogenetic activation of the GAD65 network suppressed the responses. Figure 10*D* replots the mean mouth light responses together with the taste responses from the same cells. However, for this graph, before averaging, both taste and light_m_ responses for each cell were re-ordered according to the relative magnitude of the responses to the control stimuli to yield average “tuning curves” under both conditions (note that the control responses peak at “1.0”). Interestingly, tuning curves for taste responses exhibited a much sharper peak than did the responses to the range of light_m_ frequencies tested, which declined more gradually. Thus, using light_m_ stimulation, we were able to produce firing rates resembling the output function of more broadly-tuned neurons, although the result can also be conceptualized as representing responses to increasing concentrations of a given stimulus.

We then applied linear regression to the relationship between the mean light_m_ or taste driven responses under control conditions and those suppressed by optogenetic stimulation of the NST GABA network (Fig. 10*E*). In both cases, linear functions fit the average tuning curves well (*r*^2^ = 0.995 mouth-light; 0.991, taste) and the derived functions indicated a more prominent effect on slope (mouth-light: 0.533, *p = *1.63e-04; taste: 0.371, *p = *3.61e-04) than on the Y intercept (mouth-light: 0.08, *p = *0.013; taste: −0.008, *p *= 0.458). Thus, for both mouth light and taste responses, the marked and significant deviation of the slope from unity, along with the small and, for taste, nonsignificant deviation in the intercept from zero, is consistent with the conclusion that inhibition is acting mainly in a divisive fashion.

### Behavioral effects of activating GABA neurons

**Figure 11.**
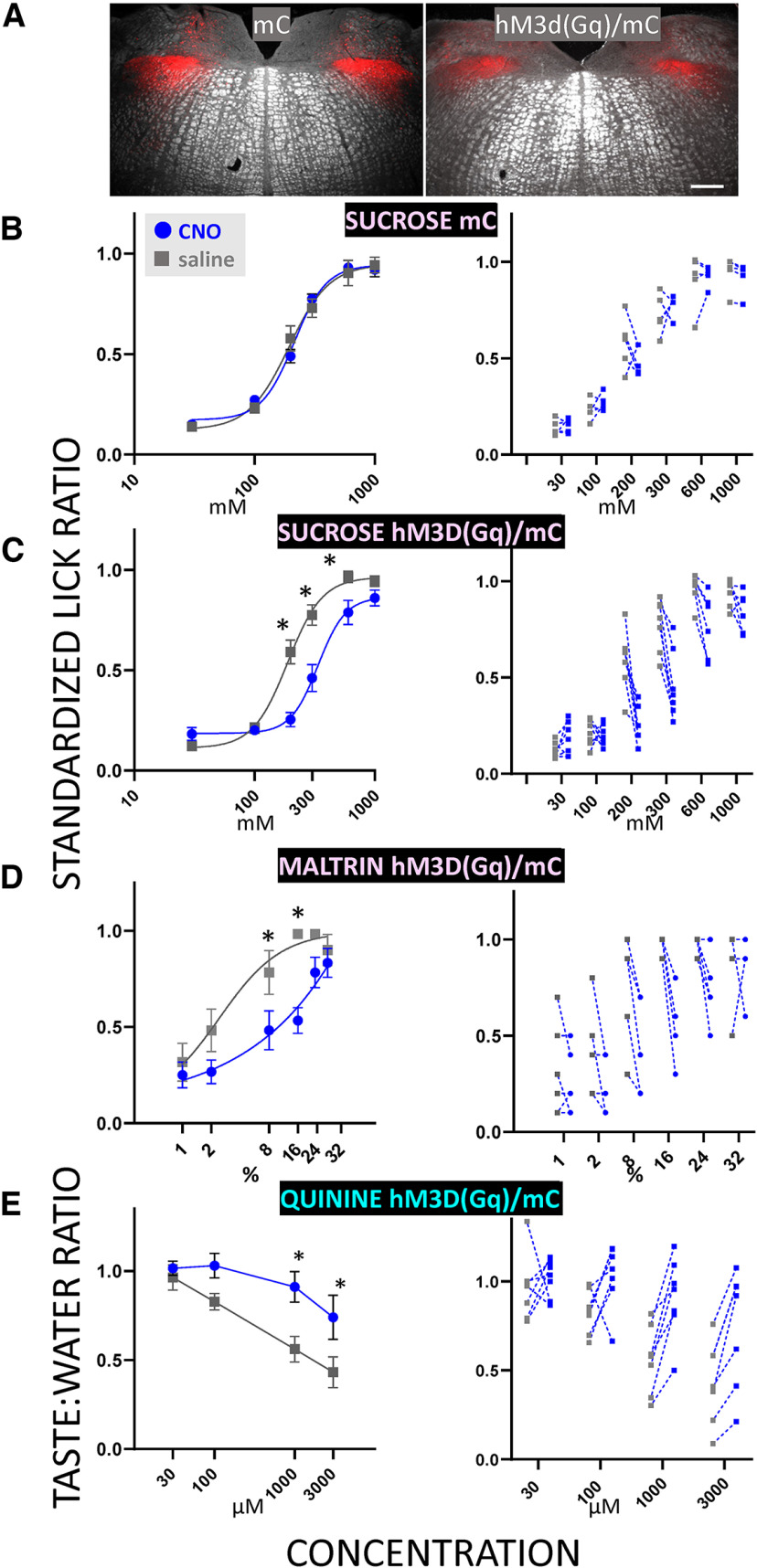
Activating rNST GAD65 neurons chemogenetically dampens behavioral acceptance of palatable (sucrose and maltrin) and rejection of unpalatable (quinine) taste stimuli. ***A***, Representative photomicrographs of mCherry (mC) expression in mice with injections of AAV2-hSyn-DIO-mCherry (mC) and AAV2-hSyn-DIO-hM3DGq/mCherry [*hM3D(Gq)/mC*] in GAD65-cre mice. Scale bar: 250 μm. ***B–E***, Behavioral effects of CNO versus saline injections in mC (*N* = 5) or hm3D(Gq)-injected mice (*N* = 8). Symbols in the left panels show mean ± SEM. Asterisks indicate significance for Bonferroni-adjusted *t* tests following ANOVA. Lines show curve fits for the equation: Y = Minimum Response + (Maximum Response − Minimum Response)/(1 + 10^((LogEC50-^*^X^*^)^*^HillSlope)^); an exception is quinine/CNO for the hM3D(Gq)/mC mice, where the function could not be fit with this equation. Right panels show data points for individual mice. The same group of eight mice was tested for all stimuli, but one or two mice were dropped from each analysis because of insufficient trials (dropped cases: GQ-13 for quinine, GQ-14 for sucrose, and both GQ14 and 7 for maltrin); therefore, the final *N*s for the three stimuli were 7 for quinine and sucrose and 6 for maltrin. ***B***, CNO had no effect on sucrose licking in mice injected with a virus that only drove expression of mC (ANOVA, drug, *p *= 0.488, concentration, *p = *9.99e-16, drug X concentration, *p *= 0.098; EC_50_ = 229 mm, saline; 230 mm, CNO). ***C***, In contrast, CNO injection decreased standardized lick ratios for sucrose (ANOVA: drug, *p *= 0.007, concentration, *p = *9.99e-16; drug X concentration: *p = *3.47e-08. Bonferroni-adjusted *t* tests: 200 mm, *p = 0.021; 300 mm, *p *= 0.021 and 600 mm; *p *= 0.042; EC_50_ = 228 mm saline; 252 mm CNO) and (***D***) maltrin (ANOVA: drug, *p *= 0.007; concentration, *p = *6.21e-10, drug X concentration, *p *= 0.004, Bonferroni-adjusted *t* tests- significant* effects of CNO for 8%, *p *= 0.021 and 16%, *p *= 0.003. EC_50_ = 2.4% saline; 6.2 × 10^6^% CNO). ***E***, Activation of GAD65-expressing neurons increased quinine licking relative to water licks for the two highest concentrations (ANOVA: drug, *p *= 0.009, concentration, *p = *7.23e-05, drug X concentration, *p *= 0.011; Bonferroni-adjusted *t* tests: 1 mm, *p = 0.028; 3 mm, *p *= 0.012). We also observed a slowing of lick rate, as reflected in the modal session ILI after CNO injection in the hM3D(Gq)/mC group during the sucrose (138 ± 5 ms CNO vs 120 ± 2.7 ms saline, *p *= 0.006) and maltrin (145 ± 5 CNO vs 119 ± 1 ms saline, *p *= 0.001) test sessions, but not during the quinine tests (123 ± 2 ms CNO vs 119 ± 1 ms saline, *p *= 0.093), which took place earlier in the test series. There were no CNO effects on lick rate in the mC control group (112 ± 3 ms CNO vs 113 ± 3 ms saline, *p* > 0.1).

Lastly, we activated GABA neurons in the rNST by expressing the excitatory DREADD, hM3D(Gq), in GAD65-expressing rNST neurons using viral injections and studied the behavioral effects on licking representative unpalatable (quinine) and palatable (sucrose and maltrin) stimuli. Injections were well-centered in the rNST (Fig. 11*A*) and largely confined to the nucleus although some spread to the overlying vestibular and underlying reticular formation occurred. No CNO effect on sucrose licking was discernable in a control group with viral injections expressing mCherry alone (Fig. 11*B*; see captions for all statistics). In contrast, licking to sucrose and maltrin decreased whereas licking to quinine was increased after CNO compared with saline injections (Fig. 1*C–E*). This is consistent with what would be expected if neural responsiveness to these hedonically opposite stimuli were both suppressed. Fitting a logistic function to the mean curves for sucrose and maltrin demonstrated a rightward shift after CNO injections. The function for quinine was unable to be fit with a logistic curve, but a rightward shift appeared to occur for this stimulus as well; rejection still occurred but required higher concentrations (e.g., compare 3000 μm with CNO to 100 μm with saline). It is notable that, in contrast to the neurophysiological effects that we observed, the behavioral effects are likely dependent mainly on local GABA neurons since this AAV virus is not transported retrogradely. Indeed, we did not observe labeling of neurons in the central nucleus of the amygdala (data not shown).

## Discussion

The prevalence of a high proportion of GABAergic neurons within the rNST, together with GABAergic terminals from extrinsic sources implies an important role for GABA in sensory processing within the nucleus. We focused on two functions for GABA: first, we established unequivocally, that a subpopulation of GABAergic neurons responds to oral gustatory stimulation. These neurons had similar gustatory profiles as identified non-GABAergic neurons, but responses were of smaller magnitude. Second the controlled optogenetic release of GABA had a largely suppressive impact on gustatory responses with only a modest sharpening of the response profile. Together, these effects did not substantially modify the ensemble pattern for taste quality. In addition, our experimental approach revealed a novel subpopulation of GABAergic interneurons that do not respond to afferent stimulation.

### Properties of rNST GABA neurons

A large number of rNST GABA neurons has been documented using immunohistochemistry (Lasiter and Kachele, 1988; Davis, 1993; M Wang and Bradley, 2010), *in situ* hybridization (Stornetta et al., 2002), and multiple reporter lines (M Wang and Bradley, 2010; Boxwell et al., 2013; S Travers et al., 2018). There are likely subclasses of solitary nucleus inhibitory neurons, as in other CNS regions (Lledo et al., 2008; Wilson et al., 2012; Burton, 2017; Kullander and Topolnik, 2021; Stachowski and Dougherty, 2021), but expression in the GAD65 cre X ChR2 mouse likely encompassed most rNST GABA cells, based on the high degree of co-localization between GAD65 and VGAT in the nucleus (S Travers et al., 2018), and comparable distributions of GAD65 and GAD67 expression in this region (Stornetta et al., 2002).

#### GABA taste neurons (G+_TASTE_)

Despite considerable effort, the present study succeeded in recording from just a small sample of G+_TASTE_ neurons. These neurons were infrequently encountered and, when detected, harder to isolate. Since anatomic studies suggest there are many GABA cells in the nucleus, we presume that their small size (Davis, 1993), and low rates of spontaneous activity partially account for the sparser sample. Despite the limitation of a restricted sample size, we were able to draw some significant conclusions.

G+_TASTE_ neurons had less robust taste responses than G-_TASTE_ cells. This is consistent with their more ventral location, which would be expected to put them in sparser contact with the more dorsal primary gustatory afferents. This observation is also consistent with *in vitro* experiments that show that GABA cells fire fewer spikes with a smaller paired pulse ratio in response to solitary tract stimulation, and that their firing rate saturates at lower levels of depolarizing current (Chen et al., 2016; Boxwell et al., 2018). The weaker responses of GABA taste neurons are also consistent with antidromic stimulation experiments demonstrating that NST taste neurons that do not project to the PBN are less responsive (Ogawa et al., 1984; Monroe and Di Lorenzo, 1995; Cho et al., 2002; Geran and Travers, 2009), implying that some non-PBN projection neurons in these studies were GABAergic. The modest number of G+_TASTE_ neurons we characterized prevented firm conclusions about more variable differences reported in antidromic studies, including a narrower breadth of tuning (Monroe and Di Lorenzo, 1995; Geran and Travers, 2009) and a propensity for more nonprojection neurons to respond to aversive stimuli (Cho et al., 2002; Geran and Travers, 2009). Each taste quality, however, activated some G+_TASTE_ (and G-_TASTE_) cells, suggesting that inhibitory neurons are not preferentially devoted to processing specific tastes.

#### GABA neurons unresponsive to taste (G+_UNR_)

We further report a novel subpopulation of GABAergic neurons unresponsive to gustatory stimulation (G+_UNR_). This lack of orosensory responsiveness suggests a different role for these neurons. On average, G+_UNR_ neurons were ventral to G+_TASTE_ neurons, which in turn were ventral to G-_TASTE_ cells. The ventral bias for GABA cells in general, and especially for G+_UNR_ neurons, is significant since this region preferentially projects to the subjacent reticular formation (JB Travers, 1988; Beckman and Whitehead, 1991; Halsell et al., 1996; Zaidi et al., 2008). This suggests that the rNST inhibitory network has strong influences on modulating autonomic and oromotor reflexes, further evident from our observations that activation of NST GABA neurons by DREADDs can influence lick rate. It may also be functionally significant that some extrinsic pathways targeting rNST, e.g., the caudal NST (S Travers et al., 2018) and central nucleus of the amygdala, preferentially contact the ventral subdivision (for review, see SP Travers and Spector, 2021). Thus, a subpopulation of G+ neurons may provide specific inhibitory functions initiated by these extrinsic inputs.

rNST neurons unresponsive to orosensory stimulation have seldom been reported, at least in anesthetized preparations. This is not surprising since the G+_UNR_ neurons we encountered had negligible spontaneous activity and were often identified only when optogenetically activated. It seems likely that these G+_UNR_ cells are activated by other CNS structures. Indeed, Smith and Li (Li and Smith, 1997) showed that suppression of rNST taste responses by electrical stimulation of gustatory cortex could be abrogated by locally infusing a GABA_A_ receptor antagonist, suggesting an excitatory pathway from taste cortex to NST GABA neurons that in turn influence taste-responsive cells. Similarly, a recent study used monosynaptic rabies tracing and optogenetic activation of the insular-NST pathway to demonstrate largely excitatory connections to rNST somatostatin neurons (Jin et al., 2021), some of which are likely GABAergic (M Wang and Bradley, 2010; Thek et al., 2019; Kalyanasundar et al., 2022). The caudal NST (S Travers et al., 2018) and central nucleus of the amygdala also project to rNST although whether they make direct connections to GABA neurons is unknown (Saha et al., 2000, 2002; Bartonjo and Lundy, 2020; Jin et al., 2021). Interestingly, some studies in the awake rat report that many rNST cells are not taste responsive, but rather are lick-rhythmic (Denman et al., 2019), although other studies did not encounter this population (Nakamura and Norgren, 1991, 1993).

### Effects of activating the rNST GABA network

Interpretation of the modulatory effects of activating the GABA network must be tempered by considering the limitations of using anesthetized mice and globally activating both local NST GAD65 neurons and extrinsic circuitry. Since isofluorane increases GABAergic tone (Brohan and Goudra, 2017) and urethane depresses spiking activity and resulting postsynaptic inhibitory currents (Sceniak and Maciver, 2006; Accorsi-Mendonça et al., 2007), it is possible an even more obvious impact of GAD65 activation would occur in awake animals. Similarly, more selective activation of subsets of GABA neurons may reveal more nuanced influences (see below). Indeed, in the natural state, the timing of inhibitory signals likely varies in a complex manner instead of occurring at a constant frequency, as we and most others have employed for optogenetic stimulation. Thus, much remains to be learned about inhibitory modulation of rNST taste responses. Nevertheless, even with these limitations, we observed that global activation of the GAD65 network produced profound suppression and that these neurophysiological effects were robust, reliable, and consistent with the impact of activating a population of rNST GABAergic neurons in behaving animals.

#### Activating the rNST GAD65 network suppresses taste responses evoked by all qualities

Optogenetically activating rNST GABA neurons and fibers markedly reduced gustatory responses of G-_TASTE_ cells and did so for all qualities and chemosensitive types of neurons. We observed no differences in the effect of GABA inhibition as a function of stimulus or chemosensitive neuron type. However, it is possible that more subtle differences would emerge with a more specific type of inhibitory manipulation or with a larger sample size. In particular, the influence of inhibition on BIT neurons requires further investigation.

*In vitro* studies in hamster and rat have shown that GABA or GABA agonists acting on GABA_A_ receptors produce increased membrane resistance and hyperpolarization in most rNST neurons that translates into decreased firing (Liu et al., 1993; L Wang and Bradley, 1993). Infusions of GABA *in vitro* at 2 mm decreased both spontaneous and afferent responses induced by anodal tongue stimulation in rNST neurons by ∼50% (Smith and Li, 1998). This decrement is comparable to the mean 44% suppression of afferent-evoked responses observed *in vitro* in GABA-neurons during optogenetic release of GABA (Chen et al., 2016) and similar to the ∼50% suppression of “best” stimulus response in the present study. Although optogenetic activation of GAD65 neurons only caused response decrements in the present study, it is possible that our strong global activation masked less prevalent GABAergic disinhibitory circuits. An *in vitro* study in the hamster demonstrated that GABA infusion caused an increase in spontaneous rate in a small proportion of neurons (Liu et al., 1993) and a recent report observed heterogeneous increases and decreases in NST taste responses using optogenetic activation of virally-driven ChR2 in GAD67 neurons (Sammons et al., 2021). Similarly, although our results demonstrate potential for GABAergic modulation of all taste qualities, more specific effects are likely in real-world situations. Two recent studies used optogenetics to suggest that amygdalar GABA projections comprise a substrate for bitter-induced suppression of sweet signals in rNST (Jin et al., 2021) or tonically inhibit bitter signaling (Bartonjo et al., 2022).

#### Activating the rNST GAD65 network has more prominent effects on gain than tuning

In addition to suppression, there was a modest sharpening of response profiles, consistent with broadening of rNST taste profiles when GABA_A_ antagonists were infused *in vivo* (Smith and Li, 1998). However, it was more impressive than the shapes of chemosensitive profiles were highly similar under inhibitory influences, suggesting that the major effect is on response gain. This conclusion was bolstered by deriving threshold linear functions that calculate slopes and *y-axis* intercepts (Semyanov et al., 2004; Atallah et al., 2012; Wilson et al., 2012; Chen et al., 2016) in the subset of cells for which we tested both taste stimuli and different frequencies of mouth lite stimulation (light_m_). This approach estimates divisive and subtractive elements of suppression and has revealed that, in some systems, different populations of GABA interneurons mainly produce one effect or the other (Semyanov et al., 2004; Atallah et al., 2012; Wilson et al., 2012; Chen et al., 2016). Divisive effects, evident in slope changes, impact neuron responses proportionally across stimulus conditions and reflect gain control, compared with subtractive effects, evident in intercept changes, which reflect a differential effect on one response over another to impact tuning. In the present study, activation of the GAD65 network changed the slope much more than the intercept, for both taste and light_m_-driven responses. These results are consistent with optogenetic inhibitory effects on the responses of rNST neurons to electrical stimulation of afferent fibers at varying frequencies *in vitro* (Chen et al., 2016). In sum, the limited effects on tuning along with the constant ensemble pattern, suggest that taste quality representation was largely unchanged, but that intensity was muted. The stable neurophysiological representation of taste quality under GABAergic challenge is further supported by the behavioral effects of activating GABA rNST neurons, where appropriate but dampened acceptance or rejection of sucrose, maltrin, and quinine occurred (Fig. 11). This is consistent with other experiments using an inhibitory DREADD to silence GAD65 neurons. In these studies, sucrose and quinine licking curves shifted to the left, suggesting that stimulus intensity was increased. However, as in the current study, the stimuli still elicited appropriate behaviors: sucrose preference and quinine rejection (S Travers et al., 2020).

### Concluding remarks

Non-GABA taste cells impacted by optogenetic release of GABA likely included glutamatergic neurons that project to PBN (Gill et al., 1999) which provides parallel inputs to the thalamocortical pathway as well as limbic structures (Norgren and Leonard, 1973; for review, see SP Travers and Spector, 2021). In addition, rNST neurons influenced by inhibition likely include those that project to the underlying reticular formation and caudal NST (JB Travers, 1988; Beckman and Whitehead, 1991; Halsell et al., 1996; Zaidi et al., 2008; S Travers et al., 2018), which are substrates for coordinating ingestion and rejection oromotor responses (Chen et al., 2001) and visceral processing. We speculate that the GABA network permits a faithful, although amplified or reduced quality message to be transmitted by the first-order gustatory relay to permit adaptive adjustments in perceptual, behavioral, and reflex responses to taste stimuli under different homeostatic states and as a function of experience.

## Synthesis

Reviewing Editor: Matthew Grubb, King’s College London Decisions are customarily a result of the Reviewing Editor and the peer reviewers coming together and discussing their recommendations until a consensus is reached. When revisions are invited, a fact-based synthesis statement explaining their decision and outlining what is needed to prepare a revision will be listed below. The following reviewer(s) agreed to reveal their identity: NONE. Note: If this manuscript was transferred from JNeurosci and a decision was made to accept the manuscript without peer review, a brief statement to this effect will instead be what is listed below.

There is very little known about the role of inhibition in gustatory processing in the rNST and CNS. This work provides an initial description of GABAergic cells in the rNST that are taste-sensitive, describes a novel population of non-taste GABA cells in ventral rNST regions that may regulate outflow, and provides insight to the role of inhibition in corresponding neural and behavioral taste response gain. This work provides important pieces of information needed to delineate brain stem taste circuits.

Generally, the stats appear correct. The only concern is the ANOVA on line 512, which has imbalanced/low group numbers (please see comments to authors). A different approach or modification may be needed.

I didn’t see one, but I think a visual abstract could be useful here, possibly showing the neural and behavioral gain effects.

In this work, Travers and colleagues combine in vivo neurophysiology, optogenetics, chemogenetics, and behavior in mice to characterize inhibitory circuity in the first synapse for taste in the mammalian brain - the rostral nucleus of the solitary tract (rNST). This structure is established to densely hold local inhibitory neurons and receive descending projections from higher nuclei that can induce inhibition. However, very little is known about the role of inhibition in gustatory processing in the rNST, including if inhibitory rNST cells respond to tastes. This work provides an initial account of the latter and describes further details on how inhibition may participate in taste processing in rNST pathways. Using single-neuron recordings and opto-tagging in mice with Cre-defined expression of ChR2 in GABAergic inhibitory neurons, the Authors identified a subpopulation of GABA-positive (G+) rNST neurons that indeed responded to tastes. G+ taste neurons had lower response rates to gustatory stimuli than non-GABA taste cells, consistent with an overall more ventral, presumably less-afferent-contact NST location also identified for G+ taste cells. G+ taste neurons responded to representatives of different taste categories, implying broad involvement of inhibition in taste quality processing. Further, the recording studies also found a novel population of taste-unresponsive G+ neurons that resided more ventrally in the nucleus, which relates to the potential functional significance of this NST region. Overall, the effect of inhibition in the rNST was on general taste response gain, more so than the gustatory tuning of taste cells. This observation was bolstered by corresponding behavioral studies that used chemogenetics to broadly silence inhibitory neurons/processes in the rNST, and found decreased and increased licking of preferred sucrose (sugar) and aversive quinine (bitter), respectively, consistent with a gain effect.

These studies are well done, with appropriate controls, and provide key details on the general effects of inhibition on taste processing. The experiments are notably very challenging, particularly making gustatory neurophysiological recordings in the mouse NTS. Like any work, there are some limitations to these studies, including the global activation of inhibition induced by the manipulations. Yet the Authors do a good job at acknowledging and discussing these and other issues, and in framing the work in the broader context of taste coding. These data, including the discovery of taste-sensitive GABAergic cells, contribute details important to delineating the organization of brain stem taste circuits, which remain in many ways still very under characterized.

I have just a few observations and suggestions, below, that aim to help improve the paper.

Major:

- There is mention early in the text that non-taste responsive GABA cells may receive centrifugal input. The rationale for this is developed later, in the discussion. But it could be helpful to the reader if this is addressed in the introduction.

- Line 512: While the authors do acknowledge the low sample size, the ANOVA is challenged by imbalanced/low group numbers, particularly for bitter cells. Analyses with BIT neurons are likely to have low power for any effect. The statement that inhibition equally impacted cell types does not clearly match the plotted results in Figure 7, which show average effects and a unique, near complete loss of activity in BIT cells under inhibition. It might be helpful to just plot all responses (by swarm plot, etc.) instead of means, which frequently don’t work well (i.e., misrepresent the center) for lower sample size neural response data. Further caution could be applied to these results and the anova interpretation/discussion.

- Line 570: more consideration should be given to the claim that the different frequencies of light-m are mimicking broad tuning, as discussed. It seems possible that a neuron could have a specified input that is variably activated across light pulse frequencies to produce the observed effects. Here, the light-m is not increasing breadth of tuning, only response frequency. Intuitively, a breadth of tuning increase would need to involve a change in circuit. More clarification and discussion of what light-m is intending to model here is needed. There does appear to be some usefulness to this technique for studying inhibitory effects, but it is unclear how light-m stimulation is mimicking anything more than rate (intensity) coding, and is very broadly doing so.

- Fig. 10E: the Y-intercepts (and slopes) shown in the equations here and discussed in the associated text don’t match the plotted functions. It would appear that the functions for taste and light-m are possibly reversed. Also, the x-axis represents taste-only, not just light-m alone, too, correct?

Minor:

- Figure 5C scale must be a typographic error, as it specifies the reticle marks 10 spikes in 500 micro-seconds (0.5 ms).

- Figure 1: please add rostro-caudal, ventral-lateral, etc. arrows to the panels in this plot, as needed, to easily orient the reader to direction in these nice images. Readers who work outside the oral cavity may not immediately recognize that in Fig. 1C, the CV/FOL image, for example, is showing a dorsal view of the tongue, and caudal is to the left.

## Author Response

Dear Dr. Grubb,

Please find our revised manuscript “Characteristics and Impact of the rNST GABA Network on Neural and Behavioral Taste Responses”. We appreciate the time and effort that you and the reviewer made to provide a thoughtful assessment.

Below, we respond in detail to each of the comments of the reviewer below. We have highlighted changes (in red) in the manuscript as well. Of most significance:

(1) As explained below, we addressed the comments regarding the ANOVA for Figure 7 by eliminating the chemosensitive group with the very small sample size (bitter cells) from the that analysis. We also replotted this Figure to include the individual data points. Overall, we come to the same conclusion; i.e., that all neuron groups (including the bitter cells) are affected similarly by optogenetic activation of the rNST inhibitory network. However, in the discussion, we take care to stress that more subtle differences could emerge with a larger sample size and that especially that “BIT” cells are worthy of further scrutiny.

(2) We changed the wording of the (single) statement in the results claiming that stimulating with mouth light (lightm) produced response profiles that “mimicked” broadly-tuned neurons. While the mouth light frequency - response curve resembled such a cell, we realize that the two phenomena are not identical. However, this procedure did give us the opportunity to efficiently test the effects of optogenetic inhibition on a different range of firing rates within single neurons, allowing more general conclusions to be made regarding inhibitory influences on input-output functions. This could be done with natural taste stimulation but would be much more time consuming and may not always be feasible due to the limited time for which single-cell isolation can be maintained. The effects of mouth light stimulation are now explained in more detail in the METHODS.

We are very glad that you and the reviewer recognized merit in our work. We trust that these additional revisions that we made (in addition to those made to the original J Neuroscience submission) will be adequate.

Sincerely,

Susan Travers

Reviewer’s Comments and our reply

Major:

- There is mention early in the text that non-taste responsive GABA cells may receive centrifugal input. The rationale for this is developed later, in the discussion. But it could be helpful to the reader if this is addressed in the introduction.

We added mention of this in the first paragraph of the introduction.

- Line 512: While the authors do acknowledge the low sample size, the ANOVA is challenged by imbalanced/low group numbers, particularly for bitter cells. Analyses with BIT neurons are likely to have low power for any effect. The statement that inhibition equally impacted cell types does not clearly match the plotted results in Figure 7, which show average effects and a unique, near complete loss of activity in BIT cells under inhibition. It might be helpful to just plot all responses (by swarm plot, etc.) instead of means, which frequently don’t work well (i.e., misrepresent the center) for lower sample size neural response data. Further caution could be applied to these results and the anova interpretation/discussion.

The reviewer makes a valid point regarding the unequal numbers of different chemosensitive types of neurons, and the very small sample of bitter cells. In response, we replotted Figure 7 and modified the statistics.

Figure 7 now includes the individual data points. However, we decided to retain a representation of the means, because an important point of the figure is to show that the shapes of the chemosensitive profiles are stable under optogenetic inhibition.

Secondly, although the “bitter” cells are retained in the plot, we removed them from the ANOVA analyses. We reran the 3-way ANOVA (light X stimulus X cell type) that examined responses across all stimuli as a function of cell type and light as well as the 2-way ANOVAs that examined absolute and percent suppression for the “best stimulus” as a function of cell type (cell type X light) sans the bitter cells. In both cases, there was still no interaction between the chemosensitive type of neuron and light, supporting the conclusion that inhibition has similar effects across neuron types. Of course, there could be more subtle differences that would emerge with more neurons or with more specific types of inhibitory manipulation (something we are currently pursuing). Regarding the “bitter” neurons, despite the small sample size, it is worth noting that activity in each of the 3 neurons examined was greatly suppressed, as seen in the plot. So, along with the behavioral results, it seems reasonable to conclude that inhibitory influences extend to bitter stimuli and this cell type. Indeed, as the reviewer points out, it is possible that effects on such cells are stronger than for others. However, it would be premature to make such a claim. We add further caveats to the DISCUSSION pertaining to the conclusion regarding the lack of differences between cell types.

- Line 570: more consideration should be given to the claim that the different frequencies of light-m are mimicking broad tuning, as discussed. It seems possible that a neuron could have a specified input that is variably activated across light pulse frequencies to produce the observed effects. Here, the light-m is not increasing breadth of tuning, only response frequency. Intuitively, a breadth of tuning increase would need to involve a change in circuit. More clarification and discussion of what light-m is intending to model here is needed. There does appear to be some usefulness to this technique for studying inhibitory effects, but it is unclear how light-m stimulation is mimicking anything more than rate (intensity) coding, and is very broadly doing so.

The reviewer makes a reasonable point. Despite the fact that the overall shape of the mouth light- response function resembles a tuning curve of broadly-tuned neuron, the underlying circuitry is unknown, and could reflect increases in responses to a given (qualitative) input, as well as recruiting additional inputs.

To provide more insight into the underlying basis for the effects of mouth light (lightm) in the GAD65-ChR2 mouse, in the METHODS (under TESTING) we now point out that the work of Breza’s lab (Baumer-Harrison et al, 2020), shows that increasing the frequency of light pulses applied to the anterior tongue elicits larger responses in the chorda tympani nerve, similar to increasing stimulus concentration. We also point out that, in single neurons, higher concentrations are known to increase firing rate in neurons most sensitive to a given stimulus as well as to recruit responses elicited by less effective stimuli; i.e., to broaden response profiles. The general rationale for using this procedure is that, because a series of mouth light frequencies can be rapidly tested, we are able to very efficiently test the effects of optogentic inhibition on responses that span a different range of firing rates. This allows more general conclusions regarding the effects of inhibition on the output functions of taste neurons to be drawn.

To acknowledge these complications, we changed the wording of the (one) statement in the RESULTS that contends that this procedure “mimics” a broadly tuned neuron and instead used the wording: “... using lightm stimulation, we were able to produce firing rates resembling the output function of a more broadly-tuned neuron, although the result can also be conceptualized as representing responses to increasing concentrations of a given stimulus.” We also changed the title of figure 10. From “Threshold linear analysis of taste and lightm responses suggests a larger effect on gain than tuning” to “Threshold linear analysis of taste and lightm responses suggests a larger divisive than subtractive effect”. In other words, we used the mathematical terms than are neutral with regard to the specifics of what the function represents.

- Fig. 10E: the Y-intercepts (and slopes) shown in the equations here and discussed in the associated text don’t match the plotted functions. It would appear that the functions for taste and light-m are possibly reversed. Also, the x-axis represents taste-only, not just light-m alone, too, correct?

Yes, they were reversed. And “taste” needed to be added to both axes. Thank you for catching these errors. They have been corrected.

Minor:

- Figure 5C scale must be a typographic error, as it specifies the reticle marks 10 spikes in 500 micro-seconds (0.5 ms).

I apologize for the confusion. These are 0.5 ms bins and the scale is for the CUMULATIVE spikes across 100 trials. We removed “/0.5ms” to avoid this confusion. The bin size is specified in the caption

- Figure 1: please add rostro-caudal, ventral-lateral, etc. arrows to the panels in this plot, as needed, to easily orient the reader to direction in these nice images. Readers who work outside the oral cavity may not immediately recognize that in Fig. 1C, the CV/FOL image, for example, is showing a dorsal view of the tongue, and caudal is to the left.

Good point; added the directions and an explanation of the viewpoint.
