# Supplementary figures and images for: Characteristics and Impact of the rNST GABA Network on Neural and Behavioral Taste Responses
Source: eNeuro. 2022 Oct 4;9(5):ENEURO.0262-22.2022. doi: 10.1523/ENEURO.0262-22.2022 (PMC9536858; doi:10.1523/ENEURO.0262-22.2022)

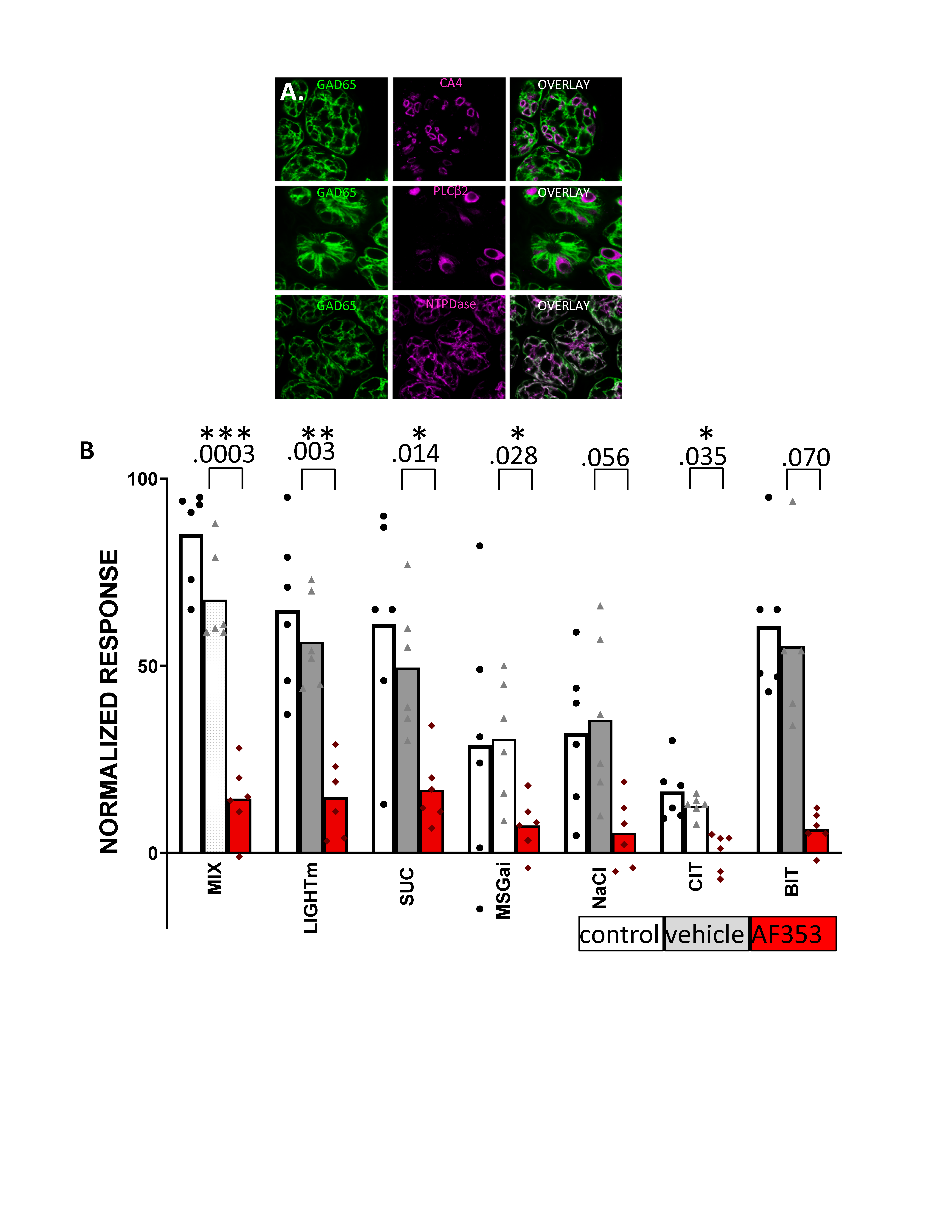

Supplement: Extended Data Figure 1-1 — Most taste bud cells in the GAD65-cre X ChR2/EYFP cross are Type I cells. Activating taste bud cells in GAD65-ChR2/EYFP mice evokes responses in NST taste neurons that are dampened by lingual application of a P2X3 antagonist. A. Immunostaining of taste buds from GAD65-ChR2/EYFP mice. Mice were perfused with phosphate-buffered saline followed by paraformaldehyde/lysine-metaperiodate and 40 μm frozen sections of fungiform and circumvallate papillae cut on a sliding microtome. Standard double-labeling immunofluorescent techniques with appropriate antibodies were used to identify Type I (NTPDase -Sevigny, host: rabbit, RRID: AB_2314986, 1:1000), Type II (PLCβ2 - Santa Cruz, host: rabbit, RRID: AB_2314986, 1:50 and Type III cells (carbonic anhydrase 4 [CA4] - R&D Systems, host: goat, RRID: AB_2070332, 1:1000). Analysis was performed by inspecting 1 μm confocal z-stacks for each of the three stains (PLCβ2 - 52 cells from 2 fungiform & 8 circumvallate papillae; CA4 - 60 cells from 4 fungiform & 6 circumvallate papillae; NTPDase - 3 fungiform & 7 circumvallate buds - individual cells not counted. None of the PLCβ2- or CA4-labeled cells co-expressed EYFP. In contrast, there was extensive co-labeling of taste bud cells stained for NTPDase suggesting that most ChR2/EYFP-expressing cells in this mouse line are Type I cells (Baumer-Harrison et al., 2020; Larson et al., 2021; Rodriguez et al., 2021). Scale bar= 10 μm. B. Ionotropic P2X3 receptors are involved in conveying mouth-light driven responses centrally to a similar degree as taste responses. Multiunit NST recordings from the GAD65-ChR2/EYFP mouse (same strain as in the main part of the study). The data is from a separate series of experiments using similar techniques to record multiunit responses to probe the role of ionotropic P2X3 receptors in conveying lightm responses centrally. Ionotropic P2X2/P2X3 heterodimers (and some homodimers) are present on primary afferent taste neurons and responsible for transmitting respon [file enu-eN-NWR-0262-22-s01.tif]
